# A single-chip, dual-gain color–NIR camera for fluorescence-guided lung and breast cancer surgery

**DOI:** 10.1038/s41377-026-02437-9

**Published:** 2026-08-21

**Authors:** Zhongmin Zhu, Brianna Hajek, Yifei Jin, Sinisha Stojanovski, Anamarija Jankulovska, Darko Angjushev, Igor Djikovski, Nexhati Jakupi, Risto Colanceski, Imran Ferati, Borce Antevski, Ilir Vela, Despot Despotovski, Natasha Tolevska Dimitrovska, Magdalena Bogdanovska Todorovska, Goran Kondov, Borislav Kondov, Shuming Nie, Sunil Singhal, Viktor Gruev

**Affiliations:** 1 Department of Electrical and Computer Engineering, University of Illinois at Urbana–Champaign, 306 N Wright St, Urbana, IL 61801 USA; 2 Institute of Pathophysiology and Nuclear Medicine, Ss. Cyril and Methodius University in Skopje, Skopje, 1000 North Macedonia; 3 Department of Thoracic and Vascular Surgery, Ss. Cyril and Methodius University in Skopje, Skopje, 1000 North Macedonia; 4 Institute of Pathology, Ss. Cyril and Methodius University in Skopje, Skopje, 1000 North Macedonia; 5 Department of Bioengineering, University of Illinois at Urbana–Champaign, 1406 W Green St, Urbana, IL 61801 USA; 6 Beckman Institute for Advanced Science and Technology, University of Illinois at Urbana–Champaign, 405 N Mathews Ave, Urbana, IL 61801 USA; 7 Department of Thoracic Surgery, Perelman School of Medicine, University of Pennsylvania, 3400 Civic Center Blvd, Philadelphia, PA 19104 USA; 8 Carle Illinois College of Medicine, University of Illinois at Urbana–Champaign, 506 S Mathews Ave, Urbana, IL 61801 USA

**Keywords:** Biophotonics, Imaging and sensing

## Abstract

Near infrared (NIR) fluorescence guided surgery can highlight tumors and sentinel lymph nodes, but most clinical fluorescence imaging systems use multi-camera architectures in which residual color–NIR misregistration can mislocalize fluorescence relative to anatomy, and weak signals often require dimming operating room lights. We developed a single-chip dual-gain scientific CMOS (sCMOS) sensor with red, green, blue (RGB), and NIR channels that provides intrinsically coregistered color reflectance and NIR fluorescence under typical operating room illumination (50–80 kLux). A backside illuminated array with division of focal plane (DoFP) pixelated interference filters achieves an input-referred read noise of 2.25 electrons and a fused dynamic range >90 dB, enabling indocyanine green (ICG) detection at ~100 pM under dark-background conditions and ~500 pM under standard 50–80 kLux operating-room illumination, with quantification over four orders of magnitude. We integrated the sensor into a surgical camera and evaluated it in 20 patients with lung cancer receiving the cathepsin-activated ICG probe VGT-309 and 35 patients with breast cancer undergoing ICG-guided sentinel lymph node biopsy. In lung cancer, lesion level sensitivity was 0.90 (18/20) and positive predictive value was 0.78 (18/23), with at least one clinically significant event in 45% (9/20) of patients. In breast cancer (90 specimens), high-gain NIR imaging improved classification of gamma probe-designated sentinel lymph nodes by node-to-background ratio (ROC AUC 0.94 vs 0.60) while preserving a white light view. These results establish a single-chip route to lights-on color and NIR guidance in the operating room.

## Abstract (web summary)

A single-chip, dual-gain color–NIR camera images visible anatomy and near-infrared fluorescence simultaneously with >90 dB dynamic range, enabling real-time fluorescence-guided lung and breast cancer surgery.

## Graphical abstract

**Figure:**
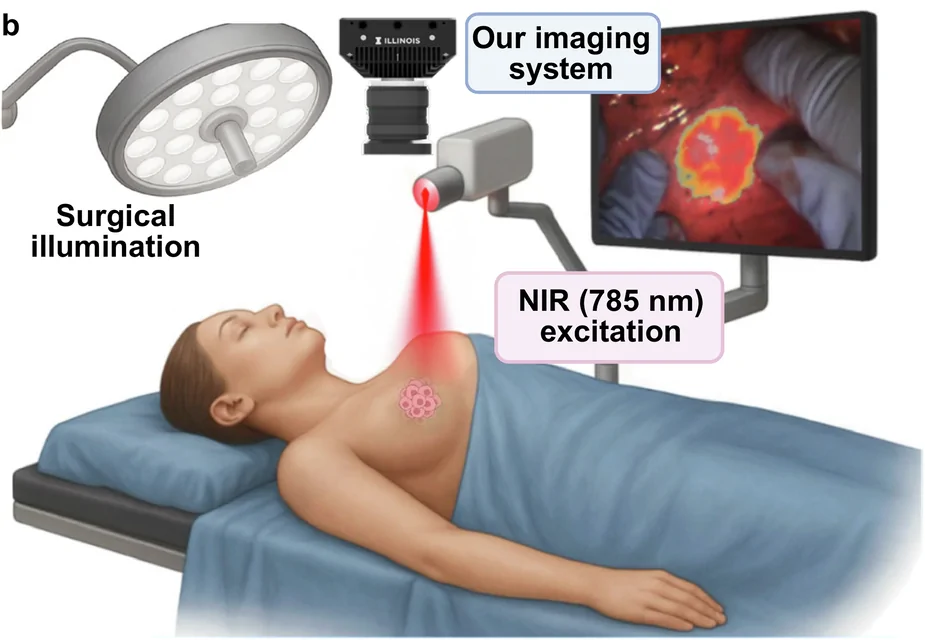

## Introduction

Silicon-based imaging sensors underpin much of modern cancer imaging, spanning preoperative modalities (e.g., X-ray imaging, computed tomography, positron emission tomography, and mammography) and intraoperative visualization platforms (e.g., fluorescence imaging and endoscopy)^1–10^. From early charge-coupled devices to modern complementary metal oxide semiconductor (CMOS) image sensors and readout electronics, continued improvements in noise, quantum efficiency, pixel density, and readout speed have expanded what can be measured in clinical workflows^11,12^. These advances have contributed to improved cancer outcomes across multiple disease sites^13,14^.

**Fig. 1:**
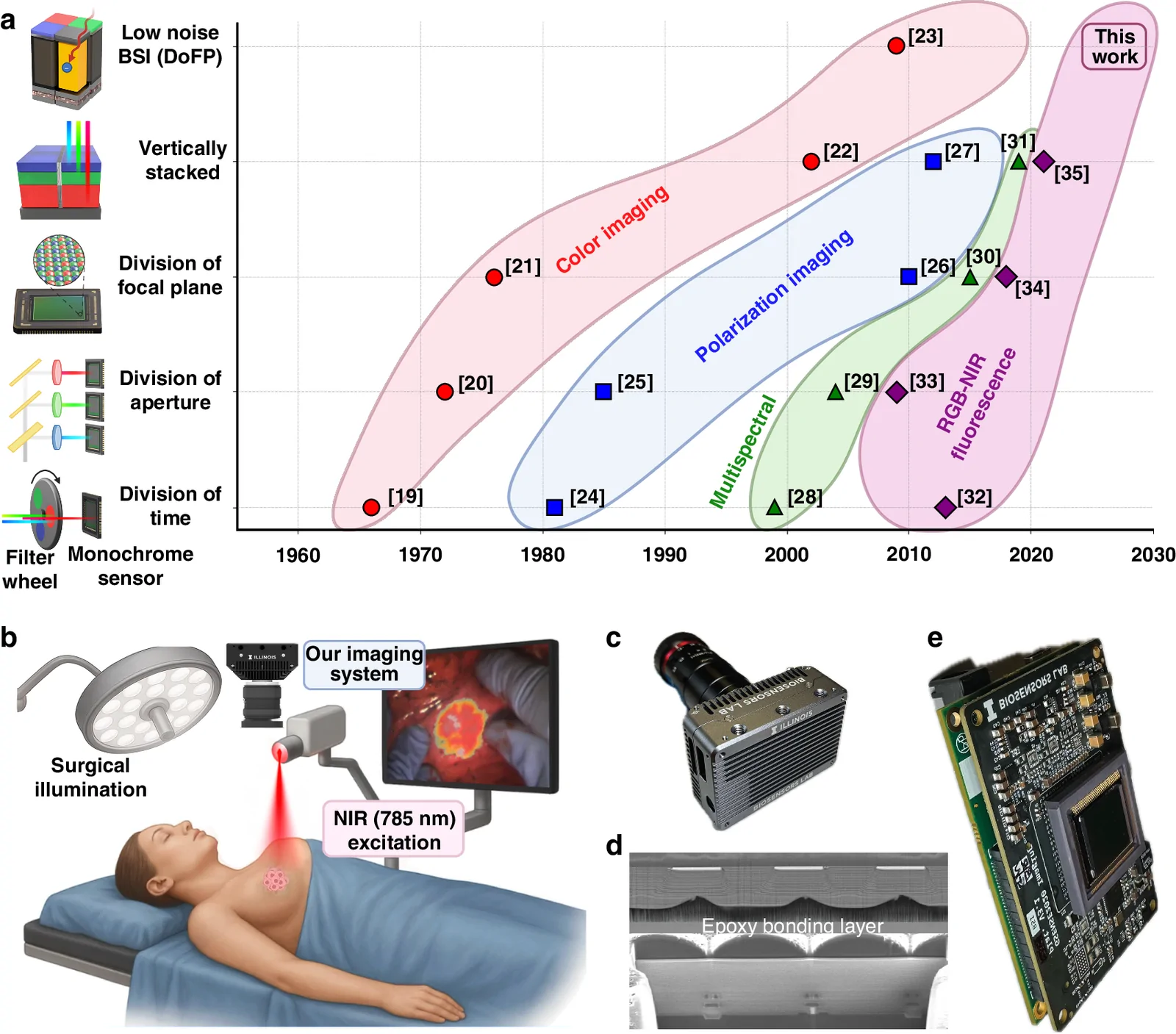
**Overview of the single-chip RGB–NIR dual-gain imaging system.** **a** Convergence map of image-sensor architectures across major imaging application domains, showing that division-of-focal-plane integration with low-noise CMOS has become the natural endpoint for color, polarization, multispectral, and (in this work) RGB–NIR fluorescence imaging. **b** Intraoperative deployment concept. **c** Packaged camera with imaging lens. **d** Cross-sectional SEM of the backside-illuminated sensor with flip-chip-bonded pixelated RGB and NIR interference filters. **e** Dual-board low-noise readout electronics

A parallel evolution in imaging has been the shift from sequential acquisition toward snapshot architectures that encode additional optical information at the pixel level, including spectrum and polarization^15–18^ (Fig. 1a). A central design question across multiple imaging fields is how to acquire spatially coregistered information from multiple optical channels—different spectral bands, polarization states, or simultaneous reflectance and fluorescence—without sacrificing signal fidelity. In each field where this question has been asked, sensor architectures have converged on the same answer: pixel-level integration of channel-selective optical elements onto a single low-noise CMOS array, an arrangement known as division of focal plane (DoFP). Color imaging completed this transition decades ago with the Bayer mosaic and modern backside-illuminated CMOS^19–21^. Polarization imaging followed once micro-polarizer arrays were monolithically integrated with imaging arrays^22,23^. Multispectral and hyperspectral imaging followed when pixelated interference-filter arrays became compatible with CMOS image-sensor fabrication^24,25^. The convergence is summarized in Fig. 1a. Fluorescence-guided cancer surgery is one imaging modality where this convergence has not yet been completed, and the clinical stakes of completing it are substantial.

Accurately identifying and completely resecting tumors during surgery remains challenging. Surgeons rely heavily on visual and tactile cues, which can be insufficient for precise margin delineation under intraoperative conditions that include variable lighting, blood and cautery byproducts, tissue deformation, and changing viewing angles. As a result, incomplete resections and positive surgical margins occur at clinically concerning rates. For example, roughly one in four women undergoing breast conserving surgery requires reoperation, most often for positive or close margins; incomplete polyp resection has been reported during colonoscopy; and oral cavity and other head and neck cancers have among the highest positive margin rates across common solid tumors, with institutional rates reaching 20–40% in some series^26–28^. These residual disease rates are associated with higher local recurrence, worse survival, and increased health care costs due to additional surgery, radiotherapy, and systemic therapy.

Fluorescence-guided surgery, particularly in the near infrared (NIR) range, has emerged as a strategy to address these limitations^9,29,30^. NIR wavelengths offer deeper tissue penetration and lower autofluorescence than visible light, improving tumor to background contrast. Regulatory approvals such as Cytalux, together with numerous ongoing clinical trials of fluorescent probes, reflect growing clinical momentum^10,31^. Yet most clinically deployed NIR fluorescence systems still rely on division of aperture architectures^32,33^ in which dichroic beam splitters route visible and NIR light to separate cameras. While effective for capturing multiple spectral bands, these multi-camera systems remain bulky and expensive, complicate sterilization and integration with surgical tools, and can be susceptible to spatial misregistration and drift over time^34,35^. For open surgery, they also struggle to detect weak fluorescence against intense surgical illumination, often prompting dimming of operating room lights and intermittent switching between white light and fluorescence views, which can disrupt workflow and reduce adoption.

We developed a single-chip, dual-gain RGB–NIR image sensor that extends the DoFP convergence principle to intraoperative molecular guidance. In its intended deployment, broadband surgical illumination and a 785 nm laser excitation source share the surgical field while the camera acquires visible reflectance and NIR fluorescence from the same field of view and generates a high-dynamic-range fluorescence overlay for the surgeon (Fig. 1b). The packaged camera (Fig. 1c) houses the sensor, supporting electronics, and the imaging lens in a compact, surgically practical form factor. At the device level, a cross-sectional scanning electron microscopy image of the backside-illuminated sensor (Fig. 1d) reveals the pixelated RGB and NIR interference filters flip-chip-bonded to the imaging array, separated by a thin epoxy bonding layer. Sensor operation and high-throughput readout are handled by two 12-layer printed circuit boards (PCBs) that integrate the low-noise dual-gain RGB–NIR sensor with the data acquisition hardware (Fig. 1e). This monolithic integration of pixelated spectral filters with the imaging array is what enables the intrinsic spatial coregistration between the visible and NIR channels. Dual-gain readout provides low-noise detection of weak fluorescence while preserving a simultaneous white light view under standard operating room illumination (50–80 kLux in our operating rooms). We evaluate the system in two clinical settings: lung cancer surgery using a cathepsin-activated indocyanine green (ICG) probe (VGT-309) and ICG-guided sentinel lymph node biopsy (SLNB) in patients with breast cancer.

## Results

### Photon-flux constraints in intraoperative fluorescence imaging

**Fig. 2:**
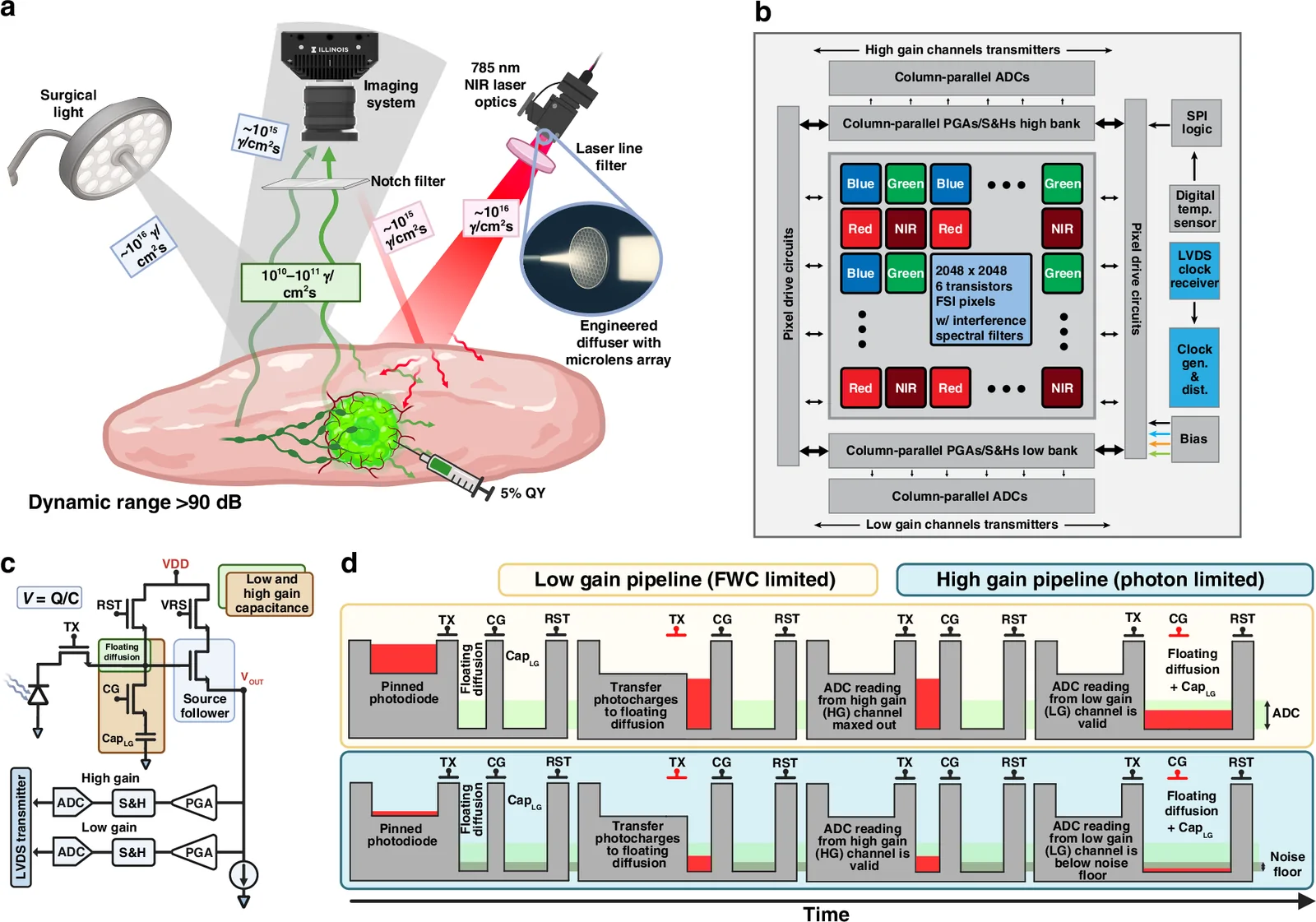
**System-level architecture and pixel operation of the dual-gain color and NIR fluorescence imaging platform.** **a** Schematic of the intraoperative imaging geometry in which bright surgical illumination is combined with focused 785 nm excitation. Fluorescence emitted from dye-labeled tissue is collected together with reflected white light, producing photon fluxes that span several orders of magnitude and require at least 90 dB of sensor dynamic range. **b** Block diagram of the 2048 × 2048 six-transistor backside illuminated array with patterned interference spectral filters and dual column parallel readout paths. Dedicated high-gain and low-gain amplifier and analog-to-digital converter banks operate concurrently on every color and NIR pixel. **c** Circuit schematic of the split capacitance pixel in which charge from the pinned photodiode is transferred to a floating diffusion that can be connected either to a small sense capacitor (high gain, low noise) or to a larger capacitor (low gain, high full well capacity) before buffering to the column circuitry. **d** Timing diagram for the low-gain (full well limited) and high-gain (photon-limited) pipelines, illustrating how the two readouts are interleaved within a single exposure and subsequently fused to generate high dynamic range (HDR) images that avoid saturation and retain sensitivity to weak fluorescence. Both readouts occur within a single exposure period rather than across alternating exposures, so dual-gain operation does not reduce frame rate

The goal of NIR fluorescence-guided cancer surgery is to provide surgeons with simultaneous visible light and NIR fluorescence images so that cancerous tissue can be precisely removed while preserving healthy tissue. Achieving this dual imaging capability requires illumination of the surgical field with both visible and NIR light and an image sensor that can reliably detect photons across these bands under operating room conditions (Fig. 2a). In the United States, surgical luminaires are typically evaluated against the International Electrotechnical Commission (IEC) standard IEC 60601-2-41. This standard, together with similar consensus standards recognized by the U.S. Food and Drug Administration (FDA), specifies that surgical lights provide 40–160 kilolux (kLux) central illuminance, a correlated color temperature of 3000–6700 K, and a color rendering index *R*_*a*_ ≥ 85 to support accurate tissue visualization^36^. In our operating rooms, central illuminance at the surgical field was measured at 50–80 kLux, depending on whether one or two surgical light heads were used.

For NIR excitation, laser-based sources are preferred over light-emitting diodes (LEDs) because they can deliver higher power with a narrow spectral bandwidth, which is important when imaging fluorophores with small Stokes shifts. At 785 nm, the maximum permissible exposure is on the order of 150 mW/cm^2^ without causing thermal damage to tissue. In this study, 785 nm excitation was delivered at an irradiance of 20 mW/cm^2^ without the use of protective laser eyewear.

An image sensor designed for fluorescence guided surgery must simultaneously capture photons from both the visible and NIR bands, even though the photon fluxes in these channels differ by several orders of magnitude (Fig. 2a). Under 50–80 kLux of visible illumination, on the order of (5–8) × 10^16^ photons/cm^2^ s reach the surgical field. Given the reflective properties of human skin and soft tissue, roughly (1–2) × 10^15^ photons/cm^2^ s are reflected toward an imaging system positioned about 1 m above the patient and form the white light image^37^.

For NIR excitation at 785 nm with a laser irradiance of 20 mW/cm^2^, about 8 × 10^16^ photons/cm^2^ s are incident on the tissue, of which ~4 × 10^15^ photons/cm^2^ s are specularly reflected and do not carry fluorescence information. These reflected NIR photons must be strongly attenuated by high optical density spectral filters in front of the sensor. The remaining photons penetrate the tissue, where they undergo scattering and absorption before exciting tumor-targeted fluorophores.

Assuming a quantum yield of 5% and a local fluorophore concentration of 10 μM, approximately 2 × 10^13^ fluorescent photons/cm^2^ s are emitted. After multiple scattering and absorption events, only on the order of 6 × 10^10^ fluorescent photons/cm^2^ s emerge from the tissue surface and are available to be captured by the sensor. Comparing visible reflectance (~10^15^ photons/cm^2^ s) to emergent NIR fluorescence (~10^10^–10^11^ photons/cm^2^ s) shows that the sensor must accommodate between four and five orders of magnitude in photon flux, corresponding to a required dynamic range of at least 90 dB. For deeper tumors or lower fluorophore concentrations, the disparity between visible reflectance and NIR emission increases, so any practical intraoperative color and NIR imaging system must provide very low read noise in the fluorescence channel together with a dynamic range of at least ~90 dB.

Guided by these photon-flux constraints, we implemented a single-chip RGB–NIR sensor with a dual-gain backside-illuminated pixel architecture. The 2048 × 2048 array uses pixelated interference filters arranged in 2 × 2 superpixels of blue, green, red, and NIR pixels, with parallel high-gain and low-gain column readout paths operating concurrently on every pixel.

### Dual-gain pixel implementation in color and NIR imaging sensors

Division of Aperture Imaging Systems addresses this dynamic range challenge by using separate cameras: one optimized for bright white light imaging with short exposure times and another optimized for low noise NIR detection with longer exposures. Single-chip color and NIR DoFP systems instead must realize both functions within a single sensor. State of the art color CMOS image sensors achieve this using per pixel dual conversion gain circuitry with separate readout paths for low-gain and high-gain signals, and we adopt the same strategy in our color and NIR sensor (Fig. 2b). For each exposure, every pixel produces two measurements: a high gain, low noise output optimized for low photon flux and a low-gain output with large full well capacity for high photon flux. Depending on the local signal level, either channel can be used alone, or the two can be fused to synthesize an HDR image.

Both the high-gain and low-gain outputs are produced concurrently from the same exposure by parallel column-readout paths (Fig. 2b–d); there is no frame-to-frame gain switching, and temporal sampling is not reduced by gain alternation. The two outputs are available for every frame and can be used individually or fused into a calibrated linear HDR image, with the full HDR fusion procedure (gain-conversion calibration, piecewise fusion rule, and display-side rendering) described in Supplementary Section S1.

Both color and NIR pixels share the same six-transistor backside illuminated (BSI) pixel circuit (Fig. 2c), which comprises a pinned photodiode (PD), transfer gate (TX), floating diffusion capacitance (*C*_FD_), a lateral overflow switch and lateral overflow capacitance (*C*_OFL_), a source follower, a reset transistor (RST), and a row select switch. The imaging array is organized into 2 × 2 superpixels comprising blue, green, red, and NIR sensitive pixels created by depositing pixelated interference filters on top of individual photodiodes. One row alternates blue and green pixels, and the adjacent row alternates red and NIR pixels (Fig. 2b). Because the blue, green, and red pixels receive relatively high visible photon flux, they typically rely on the low-gain output with large full-well capacity, whereas the NIR pixels experience much lower photon flux and therefore primarily use the high-gain, low-noise output.

Figure 2d illustrates the timing of the dual-gain readout. Each row integrates photons for a defined exposure time, after which all pixels in the row are read out in parallel. During exposure, photogenerated charge accumulates in the pinned photodiode. For high flux pixels, such as visible pixels under surgical lighting, the charge can exceed the capacity of the photodiode and overflow first into the floating diffusion and then into the lateral overflow capacitor. For low flux pixels, such as NIR pixels detecting fluorescence, charge remains stored in the photodiode.

At the start of readout, the transfer gate is pulsed, which lowers the potential barrier between the photodiode and the floating diffusion. If the photodiode has not overflowed, the stored charge is transferred onto the smaller *C*_FD_, yielding a voltage amplification proportional to the ratio *C*_PD_/*C*_FD_ (approximately 10: 1 in our design). This amplified voltage is then buffered by the source follower and digitized by the high-gain readout chain, which includes a programmable gain amplifier, sample and hold, and an analog-to-digital converter (ADC). Because the signal is amplified in pixels before encountering the column readout electronics, the input referred noise is minimized, which is essential for reliably detecting weak NIR fluorescence signals.

Next, the lateral overflow switch is enabled together with the transfer gate. If a charge overflow occurred during exposure, this step completes the redistribution of charge among the photodiode, floating diffusion, and lateral overflow capacitors, and the resulting voltage, now stored on a much larger effective capacitance, is read out through the source follower into the low-gain readout chain. This mode provides a large full well capacity for bright visible scenes at the cost of higher read noise. If no overflow occurred, the redistribution reduces the node voltage, in which case the low-gain measurement is discarded, and only the high-gain data are used.

Finally, the pixel is reset, and both the high-gain and low-gain signals are sampled again to perform correlated double sampling. This cancels *kT*/*C* reset noise, compensates for threshold variations in the source follower, and suppresses 1/*f* noise.

### Optoelectronic evaluation

**Fig. 3:**
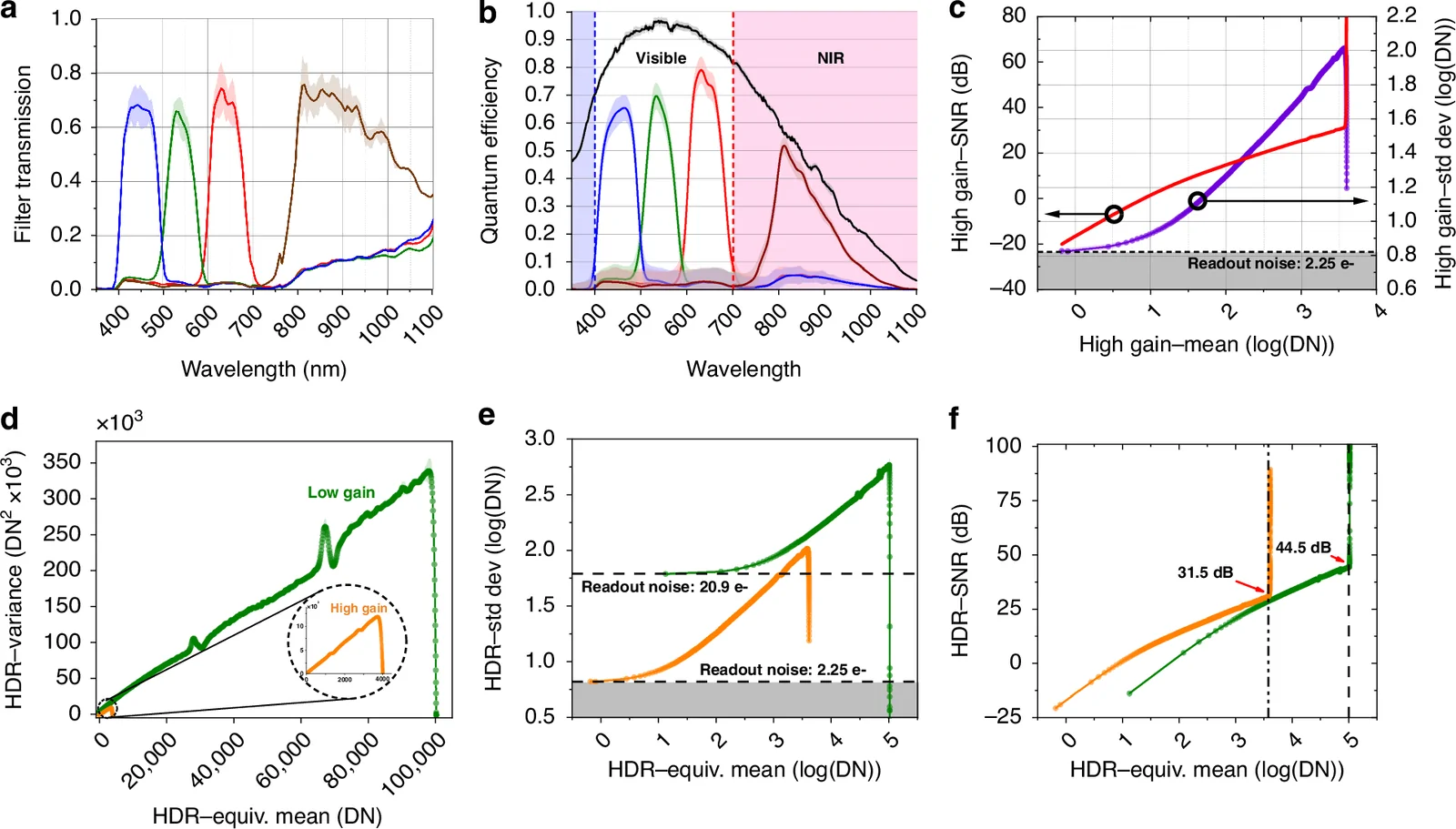
**Spectral response and noise performance of the color and NIR dual-gain sensor.** **a** Measured transmission spectra of the blue, green, red, and NIR interference filters, showing narrow visible passbands with peak transmission of ~70–75% for the color channels and high in-band transmission with strong visible rejection for the NIR channel. **b** Effective QE of the backside illuminated sensor before (black) and after integration of the pixelated filters, illustrating high QE in the visible range and a dedicated NIR band suitable for common fluorescence dyes. **c** Photon transfer analysis of the high-gain channel (standard deviation and signal to noise ratio (SNR) versus mean), highlighting the transition from read noise-limited to shot noise-limited operation and an input-referred read noise of ~2.25e^−^. **d** HDR variance versus equivalent signal for the fused high-gain and low-gain measurements, with the inset showing the corresponding high-gain-only behavior. **e** HDR standard deviation versus signal, from which read noises of ~2.25e^−^ (high gain) and ~20e^−^ (low gain) are extracted. **f** HDR SNR versus signal, demonstrating low noise at the dark end, preservation of the large full well capacity of the low gain channel, and a dynamic range exceeding 90 dB, enabled by the smooth handoff between gain channels

Pixelated interference filters for the blue, green, red, and NIR channels were fabricated on silica wafers (Torrent Photonics, Largo, FL, USA; formerly Salvo Technologies) using a four-step process that was repeated for each spectral band (Supplementary Section S2). Alternating layers of high and low refractive index dielectrics were deposited by chemical vapor deposition, followed by lithography and lift-off to define the pixel pattern. The number and thickness of layers were tuned to set the desired passband and out-of-band blocking. The measured transmission spectra of the four filter types are shown in Fig. 3a. The blue, green, and red filters define distinct bands in the visible regime with peak transmission of ~70–75%. They exhibit only weak transmission in the NIR, with an average of ~10% between 800 and 900 nm, where many clinically relevant fluorophores emit. Because the photon flux in the visible band is orders of magnitude higher than in the NIR (Fig. 2a), this small NIR leakage into the color pixels has a negligible effect on color rendering. The NIR filter was optimized for high in-band throughput, achieving >70% transmission above ~785 nm while providing visible band rejection exceeding an optical density (OD) of 4. We characterized filter transmission, quantum efficiency (QE), and noise performance (Fig. 3).

After fabrication and characterization, the filter wafer was actively aligned and flip-chip bonded onto the CMOS image sensor using a custom alignment stage developed at the University of Illinois. The resulting QE of the assembled sensor, which combines the unfiltered device and the pixelated filter stack, is shown in Fig. 3b. For reference, the QE of the unfiltered sensor is also plotted, with peak QE values of ~ 70% in the green and ~60% at 800 nm. The overall QE in each channel is reduced according to the corresponding filter passband. The NIR pixels reach QE values of ~50% at 800 nm, while the color pixels maintain high visible QE with only modest NIR sensitivity.

We next quantified the conversion gain, readout noise, full well capacity, and SNR using photon transfer measurements. For a series of uniform illumination levels, 100 frames were acquired, and the mean digital number (DN) and variance were computed for the high-gain and low-gain channels. The photon transfer curve for the high-gain channel (Fig. 3c) shows the expected transition from read-noise-limited behavior at low signal to shot-noise-limited behavior at higher signal. From the slope of the variance versus mean relationship, we extracted conversion gains of $\sim 20\,{\rm{DN}}/{{\rm{e}}}^{-}$ and $\sim 2\,{\rm{DN}}/{{\rm{e}}}^{-}$ for the high-gain and low-gain channels, respectively. Because the downstream analog chain, including the programmable gain amplifier, filtering, and ADC, is identical for both channels, this difference reflects the in-pixel gain set by the ratio of the photodiode capacitance to the floating diffusion capacitance. Extrapolating the photon transfer curves to zero signal yields input-referred read noises of ~2.25e^−^ for the high-gain channel and ~20e^−^ for the low-gain channel, with corresponding full well capacities of ~20ke^−^ and ~40ke^−^, respectively.

To evaluate the effective HDR performance obtained by fusing the two outputs, we combined the high-gain and low-gain data into an equivalent HDR signal and plotted the variance, standard deviation, and SNR as functions of this signal (Fig. 3d–f). The fused response maintains low noise at low signal while preserving the large full well capacity of the low-gain channel and reaches an overall dynamic range exceeding 90 dB.

Linearity for all four pixel types remains ~99% across the full operating range in both gain modes. Lateral resolution was measured with a USAF 1951 reflection target under the same imaging configuration used in the clinical study; using a 20% Michelson-contrast threshold, the visible channels resolved 5.66 lp/mm for the red and blue channels and 4.49 lp/mm for the green channel, corresponding to minimum resolvable half-pitches of 88.3 and 111.4 μm, respectively, and the high-gain NIR channel resolved 5.04 lp/mm under 810 nm illumination, corresponding to a 99.2 μm half-pitch. The measured depth of focus was 28.8, 27.1, 27.1, and 23.7 mm for the red, green, blue, and NIR channels, respectively. Photoresponse non-uniformity (PRNU) averaged 1.35% across channels, with a maximum of 1.51%. The sensor acquires full-frame 2048 × 2048 mosaiced images at 33 frames/s in 12-bit mode and at ~40 frames/s in 10-bit mode, with a maximum frame rate of 100 frames/s in subsampled readout. Detailed methods and channel-wise data are provided in Supplementary Sections S3–S5.

To complement these per-channel measurements with a reproducible sensor-side depth benchmark, the prototype was also evaluated against a standardized ICG-equivalent depth-sensitivity phantom (QUEL Imaging, V2_R3-03) containing calibrated fluorescent wells at eight depths up to 6 mm in a tissue-mimicking matrix with NIR optical properties matched to ICG. Under the same 785 nm excitation (20 mW/cm^2^) and imaging configuration used in the clinical study, the prototype preserved measurable contrast (baseline ratio >1) at every phantom depth, with a mean baseline ratio of 2.86 across the eight wells and 1.36 at the deepest 6 mm well (Supplementary Section S6).

The 2 × 2 RGB–NIR mosaic samples each spectral channel once per superpixel, giving a native 1024 × 1024 sampling grid per channel on the 2048 × 2048 sensor. This reduces native per-channel spatial sampling relative to a monochrome sensor of the same format, but does not reduce the integration area or readout performance of each native pixel.

Spectral crosstalk between channels is dominated by visible-band leakage into the NIR pixel, because reflected visible light from surgical illumination is orders of magnitude brighter than emergent NIR fluorescence. The pixelated dielectric interference filter on the NIR pixel suppresses visible-band transmission with an optical density greater than 4, with the in-band NIR transmission exceeding 70% above ~785 nm. A thin chromium light-blocking grid between filter elements further reduces inter-pixel optical crosstalk. The reciprocal pathway, NIR leakage into the color pixels, is small in magnitude (average ~10% transmission between 800 and 900 nm) and negligible in radiometric impact on color rendering, because visible-band photon flux dominates the color-channel signal by several orders of magnitude. No spectral-unmixing or post-acquisition crosstalk-correction algorithm was applied to the reported limit-of-detection, tumor-to-background, node-to-background, or ROC analyses; all quantitative fluorescence values were computed from dark-subtracted measured NIR-channel data.

### High dynamic range and low noise fluorescence imaging

As outlined above, intraoperative NIR fluorescence imaging imposes two stringent requirements on the image sensor. First, the system must acquire conventional color video and NIR fluorescence simultaneously, despite the several orders of magnitude difference in photon flux between visible reflections and NIR emission. Second, the sensor must detect weak fluorescence signals that are often limited by readout noise rather than photon shot noise, while still quantifying signal over a wide range of fluorophore concentrations that can span many orders of magnitude within a single field of view.

**Fig. 4:**
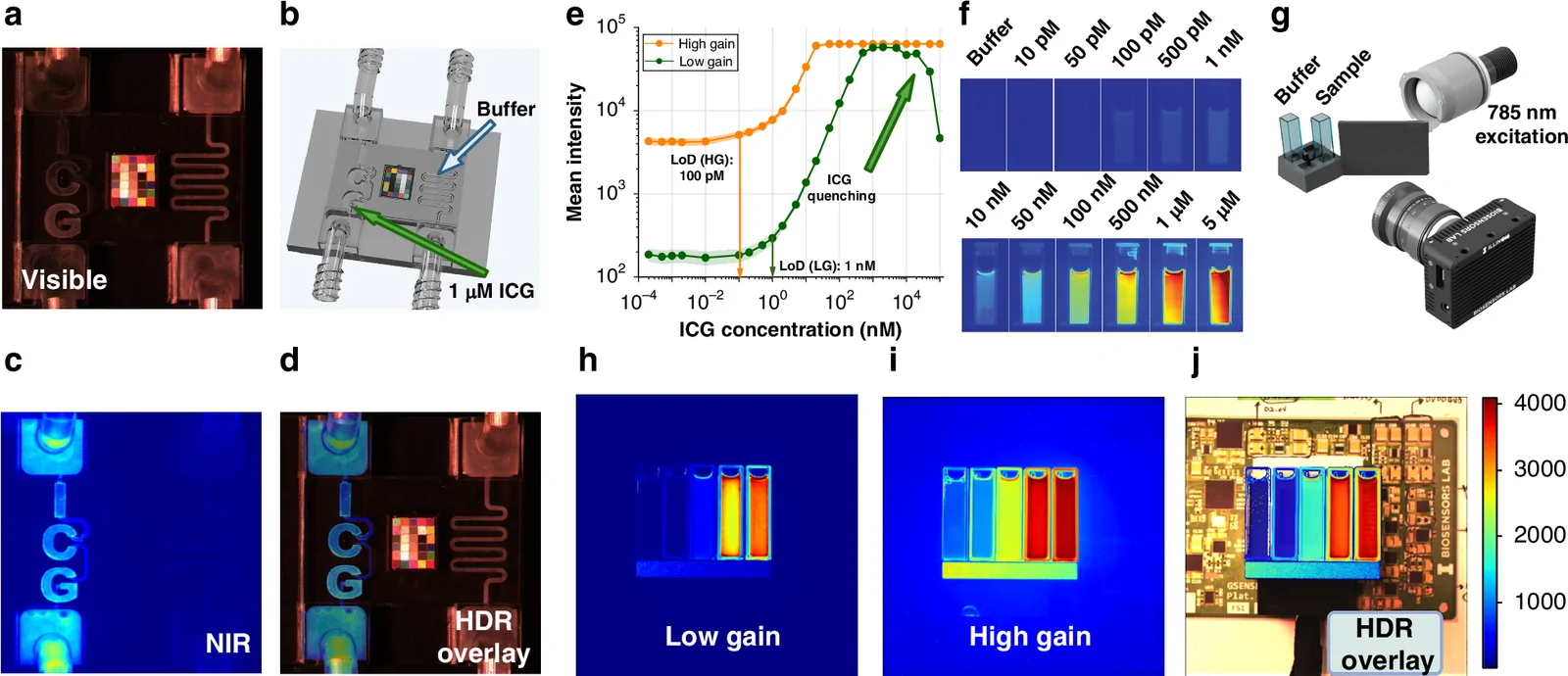
**High dynamic range color and NIR fluorescence imaging with ICG.** **a** Visible light image of a microfluidic platform containing alphanumeric and serpentine channels with a color chart. **b** Three-dimensional schematic of the device, in which one channel carries a 1 μM ICG solution, and the other carries buffer. **c** NIR fluorescence image acquired under 785 nm excitation (20 mW/cm^2^). **d** Fused HDR overlay of NIR fluorescence on the visible image, illustrating simultaneous visualization of geometry and molecular contrast. **e** Calibration curve of mean NIR intensity versus ICG concentration for the high-gain and low-gain channels, indicating limits of detection of ~100 pM and ~1 nM, respectively, and showing fluorescence quenching at high (millimolar) concentrations. **f** Cuvettes containing a dilution series of ICG, from buffer only through picomolar to micromolar concentrations. **g** Schematic of the cuvette measurement setup under 785 nm excitation (20 mW/cm^2^). **h**, **i** Example NIR images of ICG cuvettes acquired with the low-gain and high-gain channels. **j** HDR fusion of the two gain channels overlaid on a visible image of the electronics board, demonstrating that the combined HDR image can represent weak and strongly fluorescent samples over several orders of magnitude in concentration

We evaluated these capabilities using controlled ICG experiments designed to probe simultaneous color and NIR imaging and quantitative dynamic range (Fig. 4). In the first experiment, a microfluidic device incorporating serpentine channels and alphanumeric features was fabricated to create a spatially varying ICG distribution. One channel contained a 1 μM ICG solution, and the second channel contained buffer, with a color chart in the center. Under broad-spectrum illumination, the color channels capture the device geometry and the printed “ICG” pattern as a conventional color image (Fig. 4a, b). With 785 nm excitation at 20 mW/cm^2^, the NIR channel isolates fluorescence from the ICG-filled regions (Fig. 4c). Fusing the high-gain and low-gain outputs yields an HDR overlay in which variations in NIR intensity are mapped onto the white light image without loss of contextual detail (Fig. 4d).

To quantify the limit of detection (LoD) and usable dynamic range in the NIR channel, we performed a cuvette-based titration of ICG (Fig. 4e–g). Cuvettes containing a series of ICG concentrations spanning picomolar to micromolar levels were illuminated with 785 nm laser light at 20 mW/cm^2^, and both gain channels of the camera were recorded. The resulting calibration curves of mean fluorescence versus concentration (Fig. 4e) show that the high-gain channel detects ICG down to ~100 pM but saturates at low nanomolar concentrations, whereas the low-gain channel has a LoD of ~1 nM and remains linear up to millimolar concentrations before fluorescence quenching and saturation occur. The high-gain NIR channel detected ICG at ~100 pM and the low-gain channel at ~1 nM under dark-background conditions; under standard 50–80 kLux operating-room illumination, the corresponding limits of detection were ~500 pM (high gain) and ~5 nM (low-gain), with 785 nm excitation at 20 mW/cm^2^ and 25 ms exposure. LoD was defined using the IUPAC distribution-separation criterion: an ICG concentration was considered detectable when *μ*_ICG_ − 3*σ*_ICG_ > *μ*_blank_ + 3*σ*_blank_, where *μ* and *σ* are the temporal mean and standard deviation of the dark-subtracted NIR-channel signal computed across acquired frames at a fixed region of interest (ROI)^24,25^. Representative NIR images acquired in low-gain, high-gain, and fused HDR modes (Fig. 4h–j) show that the HDR reconstruction resolves weakly fluorescent cuvettes and strongly emitting samples that would individually fall below the noise floor or saturate in a single gain readout. The high-gain and low-gain NIR panels in Fig. 4h, i are displayed separately to illustrate the complementary operating regimes of the two readout paths: the high-gain channel preserves weak signals from low ICG concentrations, while the low-gain channel preserves bright signals from high ICG concentrations without saturation. In normal operation, both outputs are acquired simultaneously from every exposure (section “Dual-gain pixel implementation in color and NIR imaging sensors”) and are either analyzed individually or fused into a calibrated linear HDR image (Supplementary Section S1). Gain selection is performed after acquisition, by analyzing the appropriate linear gain channel or the fused HDR image, rather than at the time of imaging. The combined response of the two gain channels spans more than four orders of magnitude in NIR signal level, corresponding to an effective dynamic range of ~90 dB while preserving coregistered color context for surgical visualization.

### Tumor-targeted NIR imaging with VGT-309 in lung cancer surgery

To evaluate our single-chip, dual-gain color and NIR sensor with a tumor-targeted molecular imaging agent in a clinically relevant workflow, we evaluated lung cancer surgery cases in patients administered VGT-309, an activatable cathepsin-targeted probe^38,39^. This evaluation assessed whether our color and NIR camera reproduced lesion-level fluorescence classification obtained with FDA-cleared NIR instruments and whether it supported clinically actionable back-table assessments, including margin evaluation. The lung-cancer cohort was conducted as an ex vivo back-table comparison: intraoperative fluorescence imaging and surgical decision-making were performed with the FDA-cleared clinical NIR system, and the prototype RGB–NIR camera was used after resection to image the same freshly resected specimens. No surgical decisions were made using the prototype camera. This design enabled paired specimen-level comparison with the FDA-cleared workflow without altering intraoperative decision-making. Probe mechanism, dosing, and the prespecified definition of clinically significant events (CSEs) are described in “Methods.”

**Fig. 5:**
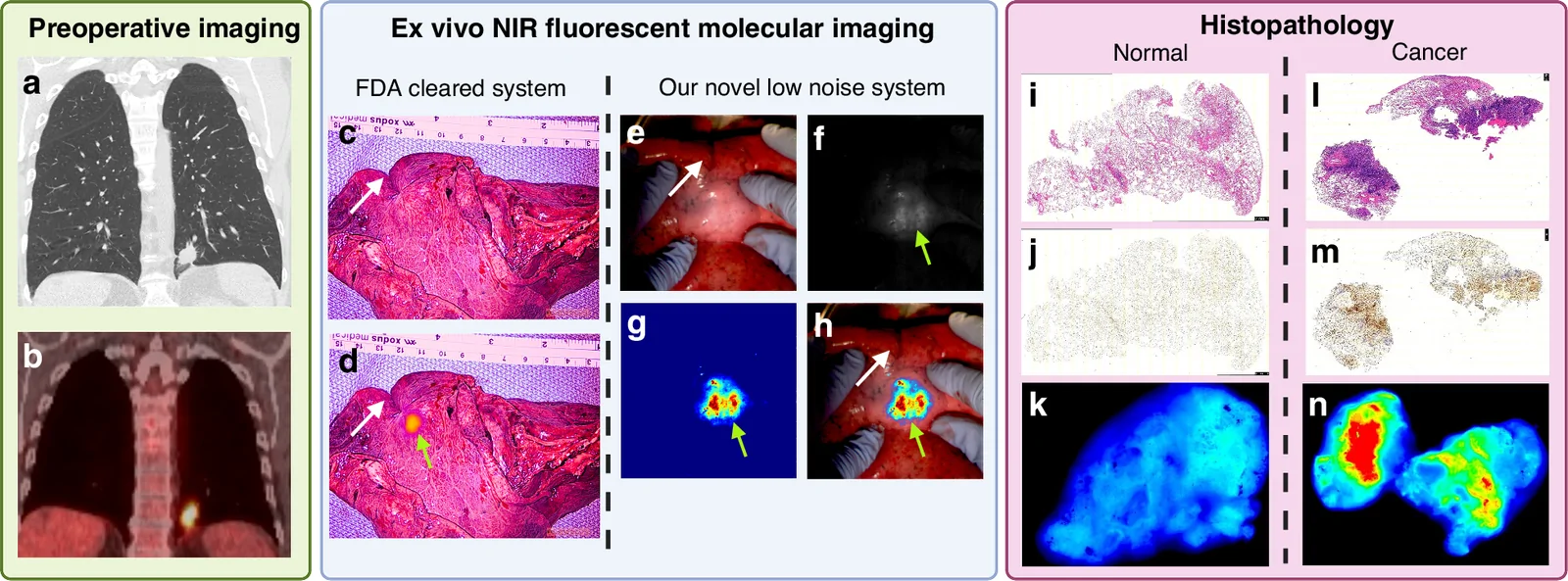
**Tumor-targeted NIR imaging with VGT-309 in lung cancer surgery using a single-chip color–NIR dual gain sensor.** Preoperative imaging of the target lesion: **a** computed tomography (CT) and **b** positron emission tomography/computed tomography (PET/CT). Ex vivo specimen imaging with an FDA-cleared NIR fluorescence system: **c** white light image and **d** corresponding fluorescence overlay. Ex vivo specimen imaging with our color–NIR dual-gain camera: **e** visible reflectance image, **f** NIR fluorescence image (high-gain channel), **g** pseudocolor NIR fluorescence heatmap, and **h** high dynamic range (HDR) overlay of NIR fluorescence on the visible image. **c**–**h** show the same resected specimen; the FDA-cleared system’s visible image was color-corrected for display, and arrows mark corresponding anatomical landmarks across the two imaging systems. **i**–**k** Normal adjacent lung and **l**–**n** tumor: hematoxylin and eosin (H& E) histology (**i**, **l**), cathepsin B immunohistochemistry (IHC) (**j**, **m**), and NIR fluorescence of adjacent sections acquired with a flatbed scanner (**k**, **n**)

We evaluated our camera in a 20-patient subset. All patients received 0.32 mg/kg VGT-309 before pulmonary resection. Pathologic stage was I in 15 patients, II in 1 patient, III in 1 patient, and unavailable in 3 patients. Surgeons performed 24 resections (wedge resection, *n* = 11; segmentectomy, *n* = 4; lobectomy, *n* = 9), removing 29 lesions in total. Preoperative imaging identified 25 of 29 lesions (Fig. 5a, b). Among the 25 preoperatively identified lesions, 16 were localized using standard methods (white light and/or palpation), whereas 9 were not localized by standard methods and were subsequently localized using NIR imaging. Histopathology confirmed malignancy in 8 of these 9 lesions and benign disease in 1.

Using the prespecified CSE definition from the parent Phase 2 VGT-309 trial^39^ (full criteria in “Methods”), 9 of 20 patients (45%; 95% confidence interval (CI): 0.26–0.66) experienced at least one CSE in this 20-patient subset. CSEs included fluorescence-guided localization when standard methods failed (8 patients) and fluorescence-defined close margins (2 patients); these event types were not mutually exclusive. In the two margin-related cases, fluorescence within 5 mm of the staple line was first identified ex vivo on the back table using our prototype camera, then confirmed ex vivo with an FDA-cleared NIR camera, and subsequently used to localize the corresponding region in vivo using the FDA-cleared system to guide additional resection. In both cases, histopathology confirmed malignant tissue within 5 mm of the staple line (close margin), consistent with the fluorescence finding.

For lesion-level performance analysis, specimen fluorescence classification was compared with histopathology as the reference standard, and all 29 lesions had complete data (20 malignant and 9 benign). Among malignant lesions, 18 fluoresced (true positives) and 2 did not (false negatives), corresponding to a sensitivity of 0.90 (18/20; 95% CI 0.70 to 0.97). Among benign lesions, 5 fluoresced (false positives) and 4 did not (true negatives), corresponding to a positive predictive value (PPV) of 0.78 (18/23; 95% CI 0.58 to 0.90). Lesion level estimates do not account for within-patient correlation and should be interpreted accordingly. All resected specimens were also imaged ex vivo with both our prototype camera and an FDA-cleared NIR camera; lesion-level-fluorescence-positive and fluorescence-negative classifications were identical between devices for all 29 lesions (Fig. 5c–h). Representative hematoxylin and eosin (H&E) histology, cathepsin B immunohistochemistry, and adjacent-section NIR fluorescence for normal lung and tumor are shown in Fig. 5i–n.

To provide a quantitative imaging comparison beyond binary classification, we calculated the tumor-to-background ratio (TBR) from prototype-camera images. TBR was 3.5 ± 1.2 across 15 lesions, consistent with the human VGT-309 TBR values reported by Kennedy et al.^38^ and the mean TBR of 4.5 at 0.32 mg/kg reported in the Phase 2 dose-ranging study^40^. The lung-cancer cohort was not designed or powered as a paired head-to-head optical benchmark against the FDA-cleared system; sensor-side detection-depth performance is reported separately on a standardized phantom (Supplementary Section S6).

### Dual-gain HDR NIR imaging enhances sentinel lymph node mapping in breast cancer

Next, to evaluate whether the low read noise and dual-gain HDR of our color and NIR sensor improve detection and quantification of weak ICG fluorescence under standard operating room illumination, we conducted a clinical study of sentinel lymph node (SLN) mapping in patients with breast cancer undergoing SLNB. Patients received tracers per institutional standard of care, including a radiocolloid (^99m^Tc-labeled human serum albumin, ^99m^Tc HSA) and ICG. Using a gamma probe and visual inspection, the surgical team identified and removed lymph nodes suspected to be sentinel nodes.

**Fig. 6:**
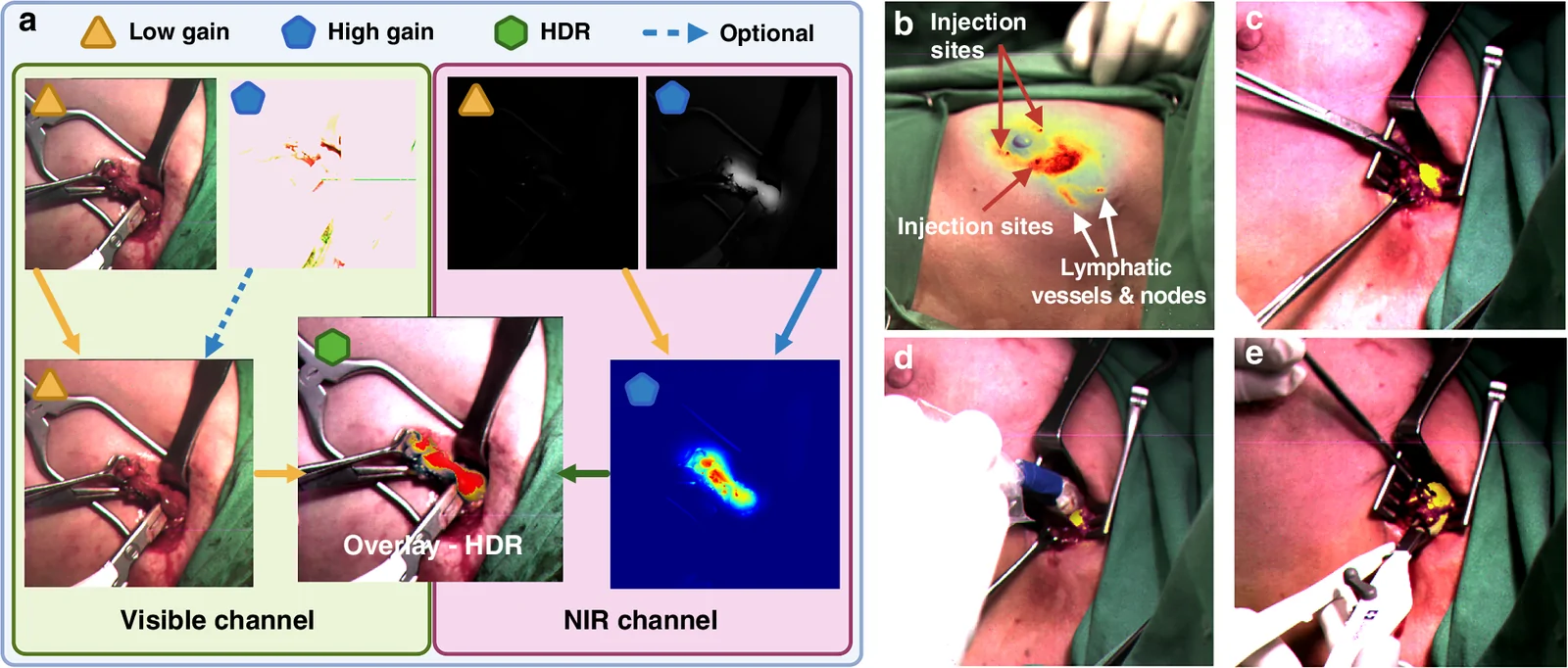
**In vivo intraoperative imaging during breast SLNB with the single-chip color–NIR dual-gain camera.** **a** Dual-gain workflow during in vivo acquisition under standard operating-room illumination and 785 nm excitation: the low-gain visible image preserves anatomical context (top left), the high-gain visible image saturates (top middle), the low-gain NIR image is dark (top right), and the high-gain NIR image reveals ICG fluorescence (bottom right), which is fused with the visible image to form the HDR overlay (bottom middle). **b** Transcutaneous ICG fluorescence imaged before incision, showing the peritumoral injection sites and a draining lymphatic vessel and node visible through the skin. **c** Intraoperative axillary view during sentinel-node localization in the surgical field. **d** Gamma-probe-correlated localization, with the gamma-probe visible in the field of view alongside fluorescent nodal tissue. **e** Direct visualization of fluorescent nodal tissue as it is grasped and removed from the surgical bed

During breast SLNB, the prototype camera acquired simultaneous visible and NIR fluorescence images directly in the surgical field under standard operating-room illumination (50–80 kLux) and 785 nm excitation (Fig. 6). The in vivo dual-gain workflow (Fig. 6a) illustrates the complementary roles of the two readout paths under live surgical conditions: the low-gain visible image preserves anatomical context, the high-gain visible image saturates, the low-gain NIR image is dark, and the high-gain NIR image reveals ICG signal, which is fused with the visible image to form the HDR overlay. Transcutaneous fluorescence from the injection sites, the draining lymphatic vessel, and the nodal region was visible before incision (Fig. 6b). Intraoperative axillary imaging during sentinel-node localization and extraction (Fig. 6c–e) shows gamma-probe-correlated localization (Fig. 6d) and direct visualization of fluorescent nodal tissue as it is grasped and removed (Fig. 6e). Real-time acquisition from the same patient is shown in Supplementary Movie 1. Fluorescence localization agreed with gamma-probe sensing and the surgeon’s visual inspection in all imaged cases.

**Fig. 7:**
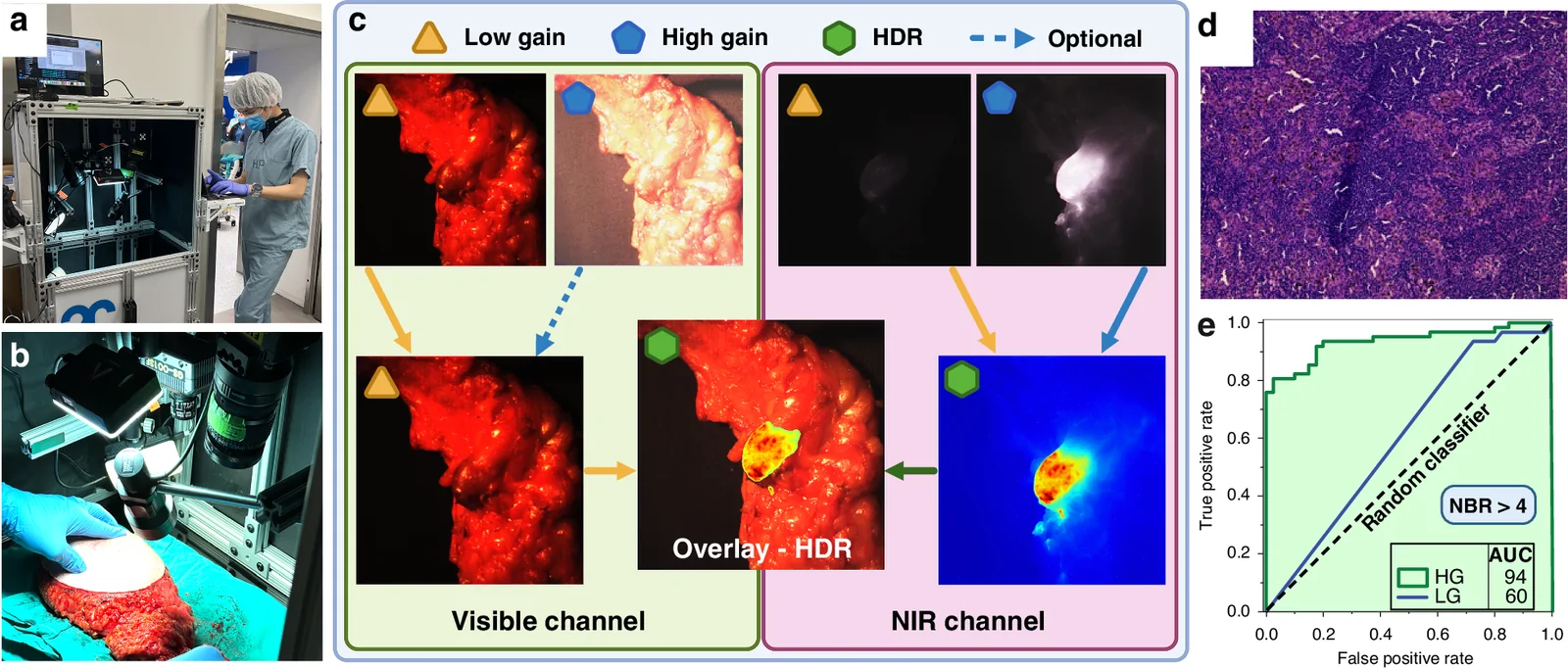
**Ex vivo back-table validation of the color and NIR dual-gain sensor during SLN mapping in breast cancer surgery.** **a** Intraoperative setup showing the imaging cart and camera used for ex vivo imaging of resected specimens. **b** Ex vivo imaging configuration for freshly resected tissue on the back table. **c** Representative images from an SLN with metastatic involvement. Left: visible channel images from the low-gain and high-gain outputs, showing that the low-gain visible image preserves anatomical detail while the high-gain image saturates. Right: corresponding NIR channel images, where the low-gain output shows minimal fluorescence and the high-gain output reveals intense ICG uptake. Middle: the fused HDR overlay maps the NIR signal onto the visible image. **d** Hematoxylin and eosin (H&E) section of a representative SLN specimen (metastasis present on histology). **e** ROC curves for the classification of gamma probe designated SLN specimens based on node-to-background ratio (NBR) measured from the low-gain and high-gain NIR channels. Sentinel designation by standard of care radiocolloid mapping with a gamma probe served as the reference standard; histopathology confirmed nodal tissue and assessed metastasis. At a nominal threshold of NBR >4, the high-gain channel achieves higher classification performance (AUC 0.94) than the low-gain channel (AUC 0.60)

Immediately after resection, all suspected nodes were submitted for histopathology to confirm nodal tissue and assess the presence of metastases. Ex vivo imaging was also performed on freshly excised specimens on the back table in the operating room (Fig. 7a, b). Using broadband illumination and 785 nm excitation, we acquired visible and NIR images from both gain channels for each specimen. Representative images from an SLN with metastatic involvement are shown in Fig. 7c. In the visible channel, the low-gain output preserves surface anatomy and texture under bright surgical lighting, whereas the high-gain visible image saturates because the increased conversion gain drives the signal toward the upper end of the dynamic range. For anatomical context, the low-gain visible image was therefore used.

In the NIR channel, the behavior is complementary. The low-gain NIR image can underestimate the ICG signal in small nodes with modest uptake, whereas the high-gain NIR image reveals a well-defined fluorescent focus corresponding to tracer accumulation. We leveraged this complementarity by retaining the low-gain visible image for structural context and the high-gain NIR image for molecular contrast, then generating an HDR overlay in which NIR intensity is mapped onto the color image (Fig. 7c, bottom panels). All suspected SLNs were either excised as discrete nodal specimens or suture-labeled within the resected tissue to enable correlation with histopathology by a board-certified pathologist.

To quantify the impact of dual-gain NIR imaging, we analyzed all resected specimens and compared fluorescence from the low-gain and high-gain NIR channels against sentinel designation by standard-of-care gamma probe mapping. Across 90 specimens, 52 were gamma-positive SLNs, and 38 were gamma-negative or non-sentinel specimens. For each specimen, ROIs were drawn over the node and over the adjacent background, and a node-to-background ratio (NBR) was computed. Specimens were classified as fluorescence positive over a range of NBR thresholds, and receiver operating characteristic (ROC) curves were generated for the two gain modes (Fig. 7e). The high-gain NIR channel improved SLN discrimination relative to the low-gain channel, with an area under the ROC curve (AUC) of 0.94 versus 0.60. Specimen-level ROC analyses treated specimens as independent observations and do not account for within-patient clustering.

The quantitative NBR and ROC analysis described above was performed on freshly resected specimens because controlled ROI placement, gamma-probe correlation, and pathology matching require standardized ex vivo conditions. Sentinel nodes varied in size, ranging from several millimeters for single uninvolved nodes to centimeter-scale conglomerates when adenopathy was present, and the prototype’s lateral resolution (88–99 μm half-pitch, Supplementary Section S3) does not limit visualization at these scales. When small sentinel nodes were embedded within larger adipose specimens, the surgeon-designated and gamma-probe-positive nodal region was suture-labeled before pathology, so fluorescence ROIs corresponded to the clinically identified nodal region rather than the whole specimen.

## Discussion

NIR fluorescence guided surgery can highlight occult tumors, margins, and SLNs, but most clinically deployed platforms rely on multi-camera architectures that split available photons across multiple optical paths and require cross-camera registration. Small residual misregistration can mislocalize the molecular signal relative to anatomy, which is clinically consequential when fluorescence targets are small and surgical decisions depend on accurate spatial correspondence to visible landmarks. Here we extend the DoFP paradigm to intraoperative color and NIR fluorescence imaging and show that a single-chip, dual-gain sensor can satisfy the combined requirements of open field surgical illumination, coregistered anatomy and fluorescence, and sensitivity to weak NIR emission.

By explicitly modeling photon flux from surgical lighting, NIR excitation, and tissue-emitted fluorescence, we established that intraoperative molecular imaging must span more than four orders of magnitude in signal while preserving performance at low photon counts. Our dual-gain backside illuminated CMOS architecture addresses this constraint by pairing low read noise in high gain with large full well capacity in low gain, yielding an effective dynamic range of ~90 dB. In contrast to multi-camera systems that require optical splitting and are susceptible to alignment drift, this platform acquires coregistered color reflectance and NIR fluorescence through a single optical path and is compatible with imaging under standard operating room lighting (50–80 kLux in our operating rooms) and 785 nm excitation at 20 mW/cm^2^.

Optoelectronic characterization and phantom studies confirm that these performance gains do not come at the expense of spectral separation or quantitative behavior. Pixelated interference filters preserve visible quantum efficiency while allocating a dedicated NIR band for clinically used ICG-based dyes, and photon transfer analysis verifies that the high-gain and low-gain outputs operate in complementary regimes. Across ICG cuvette and microfluidic phantoms, the sensor supports simultaneous color and NIR imaging over more than four orders of magnitude in fluorescence intensity. This avoids the single-gain tradeoff between saturating bright structures and losing faint signals and enables HDR overlays that retain a natural color view with superimposed molecular contrast.

The two clinical cohorts test complementary capabilities of the platform. The lung-cancer cohort, conducted as a back-table comparison against an FDA-cleared NIR system on freshly resected specimens, demonstrates that the prototype reproduces lesion-level fluorescence classification with an activatable, tumor-targeted probe (VGT-309) in a clinically practical workflow, including identification of fluorescence-defined close margins not seen by white-light inspection. The breast SLNB cohort, conducted with simultaneous color and NIR imaging under standard operating-room illumination, demonstrates that the dual-gain HDR architecture recovers weak ICG signal in small sentinel nodes while preserving anatomical context—a workflow not supported by current FDA-cleared lights-off systems. Together, these results indicate that the platform’s principal contributions are intrinsic spatial coregistration, low-noise NIR readout, and lights-on operation, rather than improvements in fluorescence visibility per se. The lung cohort was not designed or powered to establish superiority over cleared systems; the breast cohort tests classification performance under conditions that current systems cannot match.

Beyond diagnostic performance, these cohorts highlight practical advantages of a single-chip, intrinsically coregistered color and NIR design. Pixel-level coregistration eliminates parallax inherent to separated cameras and provides spatially stable overlays without per-case calibration, which is particularly important when fluorescence targets are small and surgical decisions depend on accurate localization relative to visible anatomy. The compact single lens configuration also aligns with integration into constrained form factors, including robotic, laparoscopic, and endoscopic systems, where multi-sensor solutions impose penalties in size, optical throughput, and mechanical robustness.

The architectural lineage of the present sensor traces to early backside-illuminated CMOS demonstrations such as Kohyama et al.^21^, which established small-pitch BSI for consumer color imaging using Bayer dye filters and single-gain readout. The present work extends low-noise BSI CMOS to fluorescence-guided surgery by replacing the Bayer dye filters with pixelated dielectric interference filters that include a dedicated NIR channel, increasing the pixel pitch to 6.5 μm for low-light NIR detection, and adding a dual-gain column-readout architecture optimized for weak-signal detection. Supplementary Table S1 compares the proposed camera with FDA-cleared fluorescence-guided surgery instruments, and Supplementary Table S2 compares it with representative RGB–NIR cameras. Among FDA-cleared platforms, the proposed camera is the only single-chip, intrinsically coregistered RGB–NIR system; it is the only one that operates under standard surgical illumination (50–80 kLux); and it achieves a sub-nanomolar lights-on ICG LoD (~500 pM high gain/~5 nM low gain; ~100 pM high gain/~1 nM low gain with room lights off). Among RGB–NIR cameras, the proposed sensor combines a read noise of 2.25e^−^—among the lowest reported in Table S2—with the highest dynamic range (>90 dB fused HDR) and a 100 fps maximum frame rate. The TBR of 3.5 ± 1.2 measured in our lung cohort is consistent with the human VGT-309 TBR values reported by Kennedy et al.^38^ and the mean TBR of 4.5 at 0.32 mg/kg reported in the Phase 2 dose-ranging study^40^. At the system level, the single-chip DoFP architecture also avoids the additional beam splitters, prisms, multiple sensors, and cross-camera registration required by multi-sensor surgical fluorescence systems.

The current pixelated interference-filter stack and chromium inter-pixel grid provide sufficient spectral separation for the fluorescence measurements reported here, as evidenced by sub-nanomolar lights-on ICG detection without applied crosstalk correction. Further reduction of residual leakage is achievable through higher-rejection filter-stack designs, smaller filter-to-sensor spacing, and improved optical isolation between neighboring filter elements; these are straightforward extensions of the present architecture rather than fundamental architectural changes.

The current implementation is optimized for NIR-I fluorescence around the ICG emission band and uses a single NIR channel. The RGB–NIR DoFP architecture is not intrinsically limited to a single fluorophore: because spectral response is set by a separately fabricated pixelated interference-filter array, the mosaic can be redesigned to include additional NIR-I bands matched to the emission spectra of different fluorophores, for example, by allocating multiple NIR sub-pixels within an enlarged superpixel, at the cost of reduced native spatial sampling per band. This tradeoff becomes less restrictive as sCMOS pixel pitch and pixelated interference-filter pitch continue to scale. A complementary path is to combine low-noise sCMOS readout with vertically stacked photodiode designs ^25^ to increase spectral measurements per spatial sample without reducing sampling density. The DoFP imaging architecture for one or multiple fluorescent probes is also compatible with temporal multiplexing of the excitation source^41,42^, which can further improve fluorescence sensitivity by suppressing ambient-light leakage and support multi-probe acquisition without modifications to the sensor itself. Extension to NIR-II is outside the scope of the present silicon implementation, because silicon quantum efficiency falls sharply beyond the NIR-I window; heterogeneous integration with InGaAs or analogous detectors would be required.

Conventional single-sensor color cameras already rely on mosaiced sampling and demosaicing to recover full-resolution visible images. In the present RGB–NIR layout, one red, one green, one blue, and one NIR sample are acquired per 2 × 2 superpixel; the principal penalty relative to a monochrome sensor is reduced native spatial sampling per channel, not reduced per-pixel sensitivity. The native 1024 × 1024 NIR sampling grid is well above the requirement for the specimen and sentinel-node imaging workflows evaluated here, and was reconstructed to display resolution using conventional demosaicing. CNN-based and hybrid CNN-transformer demosaicing methods we have developed for related sensor architectures^43,44^ further increase effective spatial resolution while preserving intrinsic RGB–NIR coregistration, and are a natural extension of the present sensor.

Several limitations should be acknowledged. The clinical cohorts are modest in size, and in the lung study, evaluation of the prototype camera was limited to ex vivo imaging of resected specimens, which restricts conclusions about real-time intraoperative performance for partially obscured lesions. The VGT-309 analysis was not designed or powered to establish superiority over cleared imaging systems. Instead, it supports concordance across devices and demonstrates how low noise color and NIR imaging can be paired with tumor-activated probes and margin assessment workflows. Future work can extend the filter set to additional NIR bands and integrate the sensor into clinically relevant camera heads to enable larger prospective studies that directly test whether targeted probes combined with HDR single-chip imaging reduce close or positive margins and reoperation rates. On the software side, standardized HDR visualization, quantitative node to background and tumor to background mapping, and automated segmentation may further reduce inter-operator variability and support objective decision support.

Overall, the trajectory of medical imaging has repeatedly favored single-chip designs that encode more information per pixel while simplifying optics and workflow. The results presented here suggest that intraoperative color and NIR fluorescence imaging can follow that same trajectory, enabling compact, coregistered, lights-on molecular imaging systems that are compatible with both established dyes and emerging targeted probes.

## Materials and methods

### Design of pixelated spectral filters

Pixelated spectral filters were designed using a thin-film transfer matrix model with constraints chosen to reflect practical microfabrication limits in dielectric deposition and patterning. The optimization accounted for constraints associated with chemical vapor deposition, thickness nonuniformity and surface roughness in deposited films and lithography and lift-off patterning. The optical stack was modeled with air as the incident medium and the sensor side passivation layer as the exit medium. This boundary condition reflects the fabrication flow in which the filters are fabricated on a separate silica substrate, and then flip-chip bonded onto the image sensor.

For a filter comprising *N* dielectric layers, the overall transfer matrix **A** was computed separately for s-polarized and p-polarized illumination. The corresponding matrices are

1 $${{\bf{A}}}_{s}=\left(\begin{array}{ll}{a}_{11}&{a}_{12}\\ {a}_{21}&{a}_{22}\end{array}\right)=\frac{1}{2{n}_{0}\cos {\theta }_{0}}\left(\begin{array}{ll}{n}_{0}\cos {\theta }_{0}&1\\ {n}_{0}\cos {\theta }_{0}&-1\end{array}\right)\left(\mathop{\prod }\limits_{i=1}^{N}{{\bf{M}}}_{i,s}\right)\left(\begin{array}{ll}1&0\\ {n}_{N+1}\cos {\theta }_{N+1}&0\end{array}\right)$$

2 $${{\bf{A}}}_{p}=\left(\begin{array}{ll}{a}_{11}&{a}_{12}\\ {a}_{21}&{a}_{22}\end{array}\right)=\frac{1}{2{n}_{0}\cos {\theta }_{0}}\left(\begin{array}{ll}{n}_{0}&\cos {\theta }_{0}\\ {n}_{0}&-\cos {\theta }_{0}\end{array}\right)\left(\mathop{\prod }\limits_{i=1}^{N}{{\bf{M}}}_{i,p}\right)\left(\begin{array}{ll}\cos {\theta }_{N+1}&0\\ {n}_{N+1}&0\end{array}\right)$$

where *n*_*i*_ and *θ*_*i*_ denote the refractive index and propagation angle in the *i*th medium, respectively. The layer matrices **M**_*i*,*s*_ and **M**_*i*,*p*_ for the *i*th dielectric layer are

3 $${{\bf{M}}}_{i,s}=\left(\begin{array}{ll}\cos {\beta }_{i}&-j\,\sin {\beta }_{i}\,{({n}_{i}\cos {\theta }_{i})}^{-1}\\ -j\,{n}_{i}\sin {\beta }_{i}\,\cos {\theta }_{i}&\cos {\beta }_{i}\end{array}\right)$$

4 $${{\bf{M}}}_{i,p}=\left(\begin{array}{ll}\cos {\beta }_{i}&-j\,\sin {\beta }_{i}\,\cos {\theta }_{i}\,{n}_{i}^{-1}\\ -j\,{n}_{i}\sin {\beta }_{i}\,{(\cos {\theta }_{i})}^{-1}&\cos {\beta }_{i}\end{array}\right)$$

where $j=\sqrt{-1}$, *λ* is wavelength, and *n*_*i*_ and *d*_*i*_ are the refractive index and thickness of the *i*th film. The phase thickness is ${\beta }_{i}=2\pi {n}_{i}{d}_{i}\cos ({\theta }_{i})/\lambda$. For each polarization state, the complex transmission was computed from the elements of **A** using the ratio *a*_21_/*a*_11_.

After transmission through the filter stack, photons impinge on the sensor surface and are absorbed in silicon, generating electron-hole pairs. For an incident photon flux at wavelength *λ*, denoted *F*_0_ (photons/cm^2^ s), the generation rate as a function of depth *x* follows the Beer Lambert relation

5 $$G(x)=\alpha (\lambda )\,{F}_{0}\,{e}^{-\alpha (\lambda )x}\qquad \left[{\rm{e}}-{\rm{h}}\,{\rm{pairs}}/{{\rm{cm}}}^{3}\cdot \,{\rm{s}}\right]$$

where *α*(*λ*) is the wavelength dependent absorption coefficient of silicon.

### Fabrication of an image sensor

The pixelated spectral filter array (Torrent Photonics, Largo, FL, USA; formerly Salvo Technologies) had a pixel pitch of 13 μm. Pixel-level spectral selectivity arises from a pixelated dielectric interference-filter array bonded to the backside-illuminated sCMOS sensor. Each filter type is a multilayer stack of alternating high- and low-index dielectric thin films (TiO_2_ and SiO_2_), with layer materials, thicknesses, and stack ordering specified by the transfer-matrix design framework described in the section “ Design of pixelated spectral filters.” Four spectrally distinct stacks are patterned on a fused-silica carrier wafer in a 2 × 2 superpixel layout using sequential photolithography, dielectric deposition, and lift-off, with one full lithography–deposition–lift-off cycle per filter type. A thin chromium grid between filter elements suppresses inter-pixel optical crosstalk. The full step-by-step fabrication sequence is provided in Supplementary Section S2. The CMOS image sensor (Gpixel Corporation) incorporated a pinned photodiode with low noise, dual read-out circuitry and had a pixel pitch of 6.5 μm. A custom PCB was developed at the University of Illinois at Urbana-Champaign and manufactured by PCBWay to support sensor operation and image readout at frame rates exceeding 25 frames/s. The system was integrated with an Opal Kelly data acquisition board (XEM7310) and operated using custom Verilog firmware implementing the sensor finite state machine. Control and acquisition software was developed in Python, with performance-critical functions implemented in C.

Filter to sensor alignment was performed with submicron precision using a six-degree-of-freedom positioning stage at the University of Illinois at Urbana-Champaign. The filter substrate and sensor were bonded using ultraviolet-curing optical adhesives. Because the filter pitch was twice the sensor pixel pitch, the readout firmware supported pixel subsampling to align sampled pixels to the filter pattern and to reduce data throughput to the host computer.

### NIR excitation and illumination uniformity

The 785 nm excitation source (I0785MM6000MS-7UP, Innovative Photonic Solutions) was beam-shaped using collimating optics and an engineered diffuser with a microlens array to convert the approximately Gaussian laser output into a flat-top illumination profile across the target region. The optical-power profile was measured at the imaging working distance using a calibrated photodiode power sensor (S120VC, Thorlabs) on a translation stage; spatial variation was less than 5% across the target region for a nominal central irradiance of 20 mW/cm^2^. The handheld illumination source was directed approximately normal to the tissue or specimen surface during clinical imaging. Oblique incidence reduces local irradiance through projected-area (cosine) effects and modifies tissue reflectance, so the reported irradiance and uniformity values define the intended near-normal-incidence operating geometry rather than an angle-independent property of the illumination source.

### Image reconstruction

Raw images were separated into red, green, blue, and NIR channels according to the 2 × 2 RGB–NIR mosaic, with each channel sampled on a native 1024 × 1024 grid. For visualization and overlay generation, channel images were interpolated onto the display grid using conventional demosaicing. All quantitative fluorescence analyses (LoD, TBR, NBR, ROC) were performed on dark-subtracted NIR-channel data at the native sampling grid, without any image enhancement step that could alter pixel-level intensity statistics. The HDR fusion procedure used to combine the high-gain and low-gain readouts is described in Supplementary Section S1.

### Conversion gain evaluation

Conversion gain and readout noise were measured following the EMVA 1288 standard^45^. Conversion gain was extracted independently for the high-gain and low-gain readout paths of the dual-gain image sensor. Uniform illumination was provided by an integrating sphere (819D-SF-4, Newport) driven by a 530 nm LED (M530L4, Thorlabs). The exposure time was stepped to span the usable output range while avoiding saturation.

For each exposure setting, 1000 frames were acquired. For each gain path, the mean ${\mu }_{{\rm{DN}}}$ and variance ${\sigma }_{{\rm{DN}}}^{2}$ of the digital number (DN) were computed over a central 100 × 100 pixel ROI. A photon transfer curve was constructed by plotting ${\sigma }_{{\rm{DN}}}^{2}$ versus ${\mu }_{{\rm{DN}}}$ and fitting the linear region. The fitted slope *K* corresponds to the system gain in units of DN/e^−^, and the conversion gain was reported as *g* = 1/*K* in units of e^−^/DN. The *y* intercept of the fit provides the read noise variance in DN^2^, which was converted to e^−^ using *g*.

### Quantum efficiency evaluation

Spectral quantum efficiency was characterized using a monochromator (CS260-USB-4-FH-A, Newport) coupled to an integrating sphere to provide spatially uniform, narrowband illumination from 400–1000 nm in 2 nm increments. At each wavelength, a dark stack and an illuminated stack were acquired. Specifically, 100 frames were recorded, dark subtracted, and averaged over a central 100 × 100 pixel ROI to obtain the mean signal in DN.

The optical power delivered at each wavelength was measured with a calibrated photodiode (S120VC, Thorlabs) and power meter (PM100D, Thorlabs). The corresponding photon rate was computed from the measured power and wavelength, and then converted to incident photons per pixel by accounting for the integrating sphere port geometry and the effective pixel collection area (50% fill factor). The ROI averaged DN was converted to signal electrons using the measured conversion gain *g* for the corresponding gain path. Quantum efficiency was then computed at each wavelength as

$${\rm{QE}}(\lambda )=\frac{{\rm{electrons}}\,{\rm{generated}}\,{\rm{per}}\,{\rm{pixel}}}{{\rm{incident}}\,{\rm{photons}}\,{\rm{per}}\,{\rm{pixel}}}$$

### Photoresponse non-uniformity evaluation

PRNU was quantified under spatially uniform illumination using the LED-driven integrating sphere configuration at 530 nm and 800 nm. For each wavelength, 100 frames were acquired at a fixed operating point and temporally averaged to reduce temporal noise contributions. Spatial statistics were then computed over a central 100 × 100 pixel ROI of the averaged image. Fixed pattern noise was reported as the spatial coefficient of variation,

$${\rm{FPN}}( \% )=\frac{{\sigma }_{{\rm{spatial}}}}{{\mu }_{{\rm{spatial}}}}\times 100$$

where *μ*_spatial_ and *σ*_spatial_ are the mean and standard deviation across pixels within the ROI. Detailed PRNU measurements and per-channel statistics are provided in Supplementary Section S4.

### ICG limit of detection

ICG fluorescence sensitivity was measured under two visible-light backgrounds: dark-background conditions and standard operating-room illumination measured at 50–80 kLux. In both cases, 785 nm excitation was delivered at 20 mW/cm^2^ with 25 ms exposure. ICG samples were prepared as a serial dilution in a solution of 10% fetal bovine serum (FBS) in phosphate-buffered saline spanning 10 pM to 0.1 μM, with the 10% FBS solution alone used as the blank control. For each concentration and lighting condition, 100 frames were acquired and dark-subtracted at a fixed central 100 × 100 pixel ROI. The temporal mean *μ* and standard deviation *σ* of the ROI-averaged signal were then computed across the 100 frames for both the ICG sample and the blank control, so that *σ* reflects the temporal noise floor of the sensor rather than the spatial PRNU. The LoD was defined using the IUPAC-style distribution-separation criterion: a concentration was considered detectable when *μ*_ICG_ − 3*σ*_ICG_ > *μ*_blank_ + 3*σ*_blank_.

### Sentinel lymph node mapping in patients with breast cancer

This study was conducted in accordance with the Declaration of Helsinki, under the University of Illinois at Urbana–Champaign Institutional Review Board (IRB) protocol IRB23-0314 and with approval from the Ethics Committee for Human Research, Faculty of Medicine, Ss. Cyril and Methodius University in Skopje, North Macedonia (approval No. 03-5955/8), and is registered on ClinicalTrials.gov as NCT06276439. All procedures were performed under approved guidelines, and all participants provided written informed consent. Study data were handled in a manner compliant with the Health Insurance Portability and Accountability Act.

Inclusion criteria were newly diagnosed breast cancer and age ≥18 years. Exclusion criteria included contraindications to surgery, known allergy to iodine, seafood, or ICG, uncontrolled intercurrent illness, and pregnancy or breastfeeding.

Patients received a peritumoral injection of 10 mg ICG in sterile water, followed by 3–5 min of gentle massage to promote lymphatic uptake. Patients also received radiocolloid tracer per institutional standard of care for gamma probe mapping. Breast surgery with SLNB was then performed according to standard clinical practice. During surgery, the operative field was illuminated with standard operating room lights together with 785 nm excitation, and the camera recorded color and NIR fluorescence images in vivo.

Immediately after resection, suspected SLN specimens were imaged ex vivo on the back table using the same excitation and acquisition settings. When nodes were embedded within a larger tissue specimen, sutures were used to label surgeon-designated sentinel regions to enable correlation with pathology. All excised specimens were submitted for standard clinical histopathology. H&E sections were interpreted by board-certified pathologists who were blinded to the study imaging results. Sentinel designation by standard of care radiocolloid mapping with a gamma probe served as the reference standard for classification analyses. Histopathology was used to confirm nodal tissue and assess metastasis. Image analysis, including ROI selection, was performed blinded to histopathology results.

NBR was calculated from dark-subtracted NIR images. For each suspected SLN specimen, the node ROI was drawn over the surgeon-designated and/or gamma-probe-positive nodal region, including suture-labeled regions when the node was embedded in larger adipose tissue. The background ROI was drawn over adjacent non-nodal tissue in the same specimen. Sentinel designation by standard-of-care radiocolloid mapping with a gamma probe served as the reference standard for the ROC analysis, and histopathology was used to confirm nodal tissue and assess metastasis.

### Tumor-targeted NIR imaging with VGT-309 in lung cancer surgery

This study was conducted in accordance with the Declaration of Helsinki at the Hospital of the University of Pennsylvania under IRB protocol 850893 and is registered on ClinicalTrials.gov as NCT05400226. All participants provided written informed consent. Eligible participants were adults scheduled for standard-of-care surgical resection of a lung nodule or mass suspicious for primary lung cancer or pulmonary metastasis. Key exclusions included the inability to undergo standard surgery and known allergy to ICG or other components of VGT-309. Patients received VGT-309 at 0.32 mg/kg intravenously within 12–36 h before surgery, consistent with the dose used in the parent Phase 2 protocol^39^. VGT-309 is a quenched, activity-based probe that incorporates an ICG fluorophore into a cathepsin-targeted scaffold^38^. The molecule comprises a phenoxymethyl ketone electrophile that covalently labels active cysteine cathepsins, an ICG fluorophore (excitation peak 789 nm, emission peak 814 nm), and an IRDye QC-1 quencher. Fluorescence is suppressed in the quenched state and increases after probe activation by cysteine cathepsins in the tumor microenvironment, enabling visualization on ICG-optimized NIR cameras.

CSEs were defined per the parent VGT-309 protocol^39^ and included: (i) fluorescence guided localization of a pulmonary nodule when white-light inspection and/or palpation failed; (ii) identification of a histologically confirmed synchronous lesion using NIR imaging when not detected by white-light inspection and/or palpation; or (iii) identification of a fluorescence-defined positive or close margin (<5 mm), defined as fluorescence within 5 mm of the parenchymal staple line, when the margin was judged negative under white-light inspection and/or palpation.

During the operation, the surgical team first localized and resected target lesions using standard techniques. NIR imaging was then used to localize nodules, survey for occult lesions, and evaluate resection margins. Margin assessment included inspection of the staple line and adjacent parenchyma. Fluorescence close to the staple line was treated as potentially margin-relevant and prompted targeted review of the specimen and surgical bed. Intraoperative NIR imaging was performed using the FDA-cleared clinical imaging system; the prototype camera was used for ex vivo back-table imaging of resected specimens.

TBR was calculated from dark-subtracted NIR images acquired with the prototype RGB–NIR camera. For each lesion, a tumor ROI was drawn over the fluorescence-positive lesion region, and a background ROI was drawn over adjacent non-tumor lung tissue within the same specimen. TBR was defined as the mean fluorescence intensity in the tumor ROI divided by the mean fluorescence intensity in the background ROI.

## Supplementary information

Supplementary Material

Supplementary Video

## Data Availability

The datasets and analysis code supporting the conclusions of this article are archived in the Zenodo repository at 10.5281/zenodo.18741792. The Zenodo files will be made publicly downloadable upon acceptance of this article. Raw clinical imaging data are not publicly available due to participant privacy and IRB restrictions; however, de-identified derived data and summary measurements necessary to reproduce the reported analyses will be provided via the Zenodo record.
