# Supplementary material for: A single-chip, dual-gain color–NIR camera for fluorescence-guided lung and breast cancer surgery
Source: Light Sci Appl. 2026 Aug 21;15:359. doi: 10.1038/s41377-026-02437-9 (PMC13498610; doi:10.1038/s41377-026-02437-9)
Supplement: Supplementary file 1 [file 41377_2026_2437_MOESM1_ESM.pdf]

Supplementary Information for

A single-chip, dual-gain Color–NIR camera for fluorescence guided lung and breast cancer surgery

Zhongmin Zhu[1], Brianna Hajek[1], Yifei Jin[1], Sinisha Stojanovski[2], Anamarija Jankulovska[2], Darko Angjushev[3], Igor Djikovski[3], Nexhati Jakupi[3], Risto Colanceski[3], Imran Ferati[3], Borce Antevski[3], Ilir Vela[3], Despot Despotovski[3], Natasha Tolevska Dimitrovska[3], Magdalena Bogdanovska Todorovska[4], Goran Kondov[3], Borislav Kondov[3], Shuming Nie[1,5,6], Sunil Singhal[7], Viktor Gruev[1,5,6,8*]

[1]Department of Electrical and Computer Engineering, University of Illinois at Urbana–Champaign, 306 N Wright St, Urbana, IL, 61801, USA.
[2]Institute of Pathophysiology and Nuclear Medicine, Ss. Cyril and Methodius University in Skopje, Skopje, 1000, North Macedonia.
[3]Department of Thoracic and Vascular Surgery, Ss. Cyril and Methodius University in Skopje, Skopje, 1000, North Macedonia.
[4]Institute of Pathology, Ss. Cyril and Methodius University in Skopje, Skopje, 1000, North Macedonia.
[5]Department of Bioengineering, University of Illinois at Urbana–Champaign, 1406 W Green St, Urbana, IL, 61801, USA.
[6]Beckman Institute for Advanced Science and Technology, University of Illinois at Urbana–Champaign, 405 N Mathews Ave, Urbana, IL, 61801, USA.
[7]Department of Thoracic Surgery, Perelman School of Medicine, University of Pennsylvania, 3400 Civic Center Blvd, Philadelphia, PA, 19104, USA.
[8]Carle Illinois College of Medicine, University of Illinois at Urbana–Champaign, 506 S Mathews Ave, Urbana, IL, 61801, USA.

*Corresponding author(s). E-mail(s): vgruev@illinois.edu;

This file includes:

- Supplementary Table S1. Performance comparison of representative fluorescence-guided surgery camera systems
- Supplementary Table S2. Sensor-level comparison of representative RGB–NIR camera platforms
- S1. HDR reconstruction and display rendering
- S2. Fabrication of the pixelated interference-filter array
- S3. Lateral resolution characterization (Supplementary Tables S3–S4; Supplementary Figs. S1–S7)
- S4. Fixed-pattern noise (FPN) / PRNU characterization (Supplementary Table S5; Supplementary Figs. S8–S9)
- S5. Depth of focus measurement (Supplementary Table S6; Supplementary Figs. S10–S12)
- S6. ICG-equivalent depth-sensitivity assessment (Supplementary Figs. S13–S15)
- Supplementary References

Supplementary Table S1: **Comparison of representative FDA-cleared open-field fluorescence-guided surgery instruments and the proposed prototype.** All listed clinical systems hold FDA 510(k) clearance for fluorescence imaging in humans. The proposed RGB–NIR camera is a research prototype; it is included for performance benchmarking and is not FDA-cleared.

System	Company	Sensor type	Illumination / spectral filtering	Setup	Coregistered RGB–NIR	OR lights	Fluorescence LoD (fluorophore; exposure)	Resolution (pixels)	Max FPS	ADC (bit)	Ref.
Fluobeam 800	Fluoptics (Getinge)	CCD	785 nm laser; emission filter, material NR	Single-sensor (NIR only)	No (NIR only)	Off	∼5 nM (IRDye 800CW)	720 × 576	25	8	[1]
PDE Neo II	Hamamatsu	CCD	760 nm LED; emission filter, material NR	Single-sensor (NIR only)	No (NIR only)	Off	∼15 nM (IRDye 800CW)	640 × 480	30	8	[1]
SPY-PHI	Stryker	CMOS HD	805 nm laser-diode excitation; filter material NR	Multi-sensor	Multi-sensor	Off	∼5 nM (IRDye 800CW)	1920 × 1080	60	8–12	[1–3]
Quest Spectrum	Quest Medical Imaging	CCD	LED illumination; multi-band emission filters	Multi-sensor	Multi-sensor	Off	∼10 nM (IRDye 800CW)	NR	NR	NR	[1]
VS3-Iridium	VisionSense (Medtronic)	CCD	785 nm or 805 nm laser; dual optical paths	Two-sensor	Two-sensor	Off	∼50 pM (IRDye 800CW)	960 × 720	NR	12	[1]
**Proposed RGB–NIR camera (this work)**	**University of Illinois**	**BSI sCMOS**	**Pixelated dielectric interference filters**	**Single-sensor**	**Intrinsic (single-chip)**	**On**	**ICG; 25 ms; 20 mW cm^{-2}. Lights-off: ∼100 pM. Lights-on: ∼500 pM.**	**2048 × 2048**	**100***	**10/12**	**This work**

Abbreviations: ADC, analog-to-digital converter; BSI, back-side illuminated; CCD, charge-coupled device; FPS, frames per second; HG, high gain; ICG, indocyanine green; LED, light-emitting diode; LG, low gain; LoD, limit of detection; NIR, near-infrared; NR, not reported; OR, operating room; sCMOS, scientific CMOS. "Coregistered RGB–NIR" indicates whether visible reflectance and NIR fluorescence are acquired in intrinsic spatial coregistration; "NIR only" systems do not provide a visible-channel image. "OR lights" indicates whether operating-room surgical lights must be dimmed or turned off during fluorescence acquisition; "On" indicates compatibility with standard surgical-light illumination (50–80 kLux). Reported LoD values were extracted from published literature and were not measured under a single standardized protocol; fluorophore, excitation irradiance, exposure, and detection criteria differ across systems. **Maximum frame rate of 100 fps is achieved in subsampled readout; full-frame readout is 33 fps in 12-bit mode and ∼40 fps in 10-bit mode.*

Supplementary Table S2: **Sensor-level comparison of representative RGB–NIR camera platforms.** The table emphasizes sensor metrics most relevant to low-light NIR fluorescence imaging: resolution, pixel pitch, read noise, dynamic range, frame rate, and maximum signal-to-noise ratio.

	Sensor / camera	Company / group	Resolution	Pitch	Read noise	DR	Max FPS	Max SNR	Ref.
Commercial	AR0830 RGB–NIR	onsemi	3840 × 2160	1.4 μm	3 e^-	73 dB	60	40 dB	[4, 5]
	OV2312 RGB–NIR	OMNIVISION	1600 × 1300	3.0 μm	2.2 e^-	68 dB	90	38 dB	[6, 7]
	OX05B1S RGB–NIR	OMNIVISION	2592 × 1944	2.0 μm	1.8 e^-	72 dB	60	40 dB	[8, 9]
	Mira220 RGB–NIR	ams OSRAM	1600 × 1400	2.79 μm	7.0 e^-	63.7 dB	90	36 dB	[10, 11]
	CMV4000 RGB–NIR mosaic	ams OSRAM / Spectral Devices	2048 × 2048 raw; 512 × 512/band	5.5 μm	13 e^-	60 dB	180	41 dB	[12, 13]
Research	RGB–NIR single-camera system	University of Arizona	2304 × 1296	1.85 μm	—	—	60	—	[14]
	Bio-inspired RGB–NIR imager	Washington University / University of Illinois	1280 × 720	7.8 μm	—	62 dB	40	48 dB	[15]
	Hexachromatic bioinspired camera	University of Illinois	1280 × 720	7.8 μm	62 e^-	64 dB	30	44 dB	[16]
	**Proposed RGB–NIR dual-gain sensor**	**This work**	**2048 × 2048 raw; 1024 × 1024/ch.**	**6.5 μm**	**2.25 e^- HG; ∼20 e^- LG**	**>90 dB fused HDR**	**100**	**46 dB**	**This work**

Abbreviations: ch., native per-channel sampling; DR, dynamic range; FPS, frames per second; HDR, high dynamic range; HG, high gain; LG, low gain; Pitch, pixel pitch; SNR, signal-to-noise ratio. Em dash (—) indicates data not reported. Maximum SNR is the peak signal-to-noise ratio at near-saturation, reported in dB.

Supplementary methods

Common imaging conditions. Unless otherwise noted, supplementary bench measurements were acquired under the same optical configuration used in the clinical study: the same 40 mm imaging lens (approximately 90 mm full-frame equivalent on the 18.3 mm diagonal sensor), the same working distance (∼0.5 m), the same aperture setting (f/8), and the same acquisition pipeline. Visible-channel measurements used the same broadband visible LED source employed for the visible-resolution study (Thorlabs MNWHL4, 4900 K), and NIR measurements used the same NIR LED source employed for the NIR-resolution study (Thorlabs M810L5, 810 nm).

S1 HDR reconstruction and display rendering

For each exposure, the sensor produces matched high-gain and low-gain raw images, denoted $H(x, y)$ and $L(x, y)$, from the same integration period. Following calibration under identical illumination conditions, the low-gain image was mapped into the high-gain signal domain before fusion. Let K_H and K_L denote the slopes of the linear response curves for the HG and LG channels, and let O_H and O_L denote the corresponding black-level offsets. The gain-conversion factor was defined as

$$a = \frac{K_H}{K_L}, \tag{S1}$$

and the offset-compensation term was defined as

$$b = a\,O_L - O_H. \tag{S2}$$

The low-gain image was then converted into high-gain-equivalent digital units as

$$L_{\mathrm{eq}}(x, y) = a\,L(x, y) - b. \tag{S3}$$

A linear HDR image was constructed using the HG output in the unsaturated region and the converted LG output beyond the HG linear range:

$$I_{\mathrm{HDR}}(x, y) = \begin{cases} H(x, y), & H(x, y) \le T_H \\ L_{\mathrm{eq}}(x, y), & H(x, y) > T_H \end{cases} \tag{S4}$$

where T_H was chosen near the upper end of the linear region of the HG response curve. This procedure follows the sensor-level HDR construction described for the GSENSE2020BSI platform [17].

For surgeon-facing display, the linear HDR image was subsequently converted to a human-readable rendered image using intensity normalization together with gamma/tone compression. In normalized form,

$$\tilde{I}(x, y) = \mathrm{clip}\left(\frac{I_{\mathrm{HDR}}(x, y) - I_{\min}}{I_{\max} - I_{\min}},\ 0,\ 1\right), \tag{S5}$$

and the display image was generated as

$$I_{\mathrm{disp}}(x, y) = \tilde{I}(x, y)^{1/\gamma}, \tag{S6}$$

with γ chosen to preserve visibility in both shadow and highlight regions. Additional display-side ISP operations, including overlay rendering and contrast adjustment, were used only for visualization. Quantitative analyses reported in this study were based on the linear HG, LG, or HDR image data rather than on the gamma-compressed display image.

S2 Fabrication of the pixelated interference-filter array

The four pixelated filter stacks were patterned sequentially on a transparent fused-silica carrier wafer, with one lithography–deposition–lift-off cycle per filter type. The wafer was first coated with a 20 nm chromium layer that defines an inter-pixel light-blocking grid in the final array. A photolithographic pattern was then used to open windows at the pixel locations corresponding to one filter type. The exposed chromium within these openings was removed by reactive-ion etching, exposing the fused-silica surface only at the locations of the current filter type. A sacrificial release coating was patterned in the same openings using a second photolithography step. A multilayer stack of TiO_2 and SiO_2 was then deposited across the entire wafer by physical vapor deposition, with layer thicknesses and ordering taken from the transfer-matrix design for that filter type (Section 3.1 of the main text). The wafer was immersed in resist remover with mild ultrasonic agitation; removal of the sacrificial release layer lifted off the dielectric stack everywhere except in the patterned openings, leaving a pixel-localized multilayer filter stack on the fused-silica surface. This cycle was repeated three additional times, with a different photomask and a different transfer-matrix-designed dielectric stack each time, to pattern the remaining three filter types into their assigned positions within each 2×2 superpixel.

After all four filter stacks were patterned, the completed filter wafer was flip-chip aligned and bonded to the backside-illuminated sCMOS sensor wafer using a six-degree-of-freedom positioning stage and UV-cured optical adhesive, with submicron registration between filter elements and sensor pixels across the 2048×2048 array. Filter fabrication and characterization were performed at Torrent Photonics (Largo, FL, USA; formerly Salvo Technologies). Active alignment and bonding to the sensor were performed at the University of Illinois Urbana–Champaign.

S3 Lateral resolution characterization

All measurements in this section were performed in the same optical configuration used for the clinical study: the same 40 mm imaging lens (approximately 90 mm full-frame equivalent on the 18.3 mm diagonal sensor), the same working distance (∼0.5 m), the same aperture (f/8), and the same acquisition settings used for clinical imaging. Visible-channel resolution was evaluated under white-light illumination, and NIR resolution was evaluated under 810 nm illumination. A negative USAF 1951 reflection target (Thorlabs R1DS1N1, groups 0–3) was used for all measurements.

S3.1 Experimental setup and sampled line profiles

The target was imaged at the object plane using the clinically matched optical setup described above. For visible resolution, the low-gain visible channels were analyzed under white LED illumination (4900 K). For NIR resolution, the high-gain NIR channel was analyzed under 810 nm LED illumination because that readout path is the NIR mode used for weak-signal fluorescence imaging in the main study. Supplementary Fig. S1 shows the target and representative locations of the vertical line profiles extracted for contrast analysis.

S3.2 USAF spatial frequency, five-point Michelson contrast, and pass criterion

For a USAF 1951 target, the spatial frequency corresponding to group G and element E is

$$f(G, E) = 2^{G+(E-1)/6} \quad \mathrm{lp\,mm^{-1}}. \tag{S7}$$

For each selected line profile, three bright samples (high points) and two dark samples (low points) were picked across the three-bar pattern of the corresponding element. Let H_1, H_2, and H_3 denote

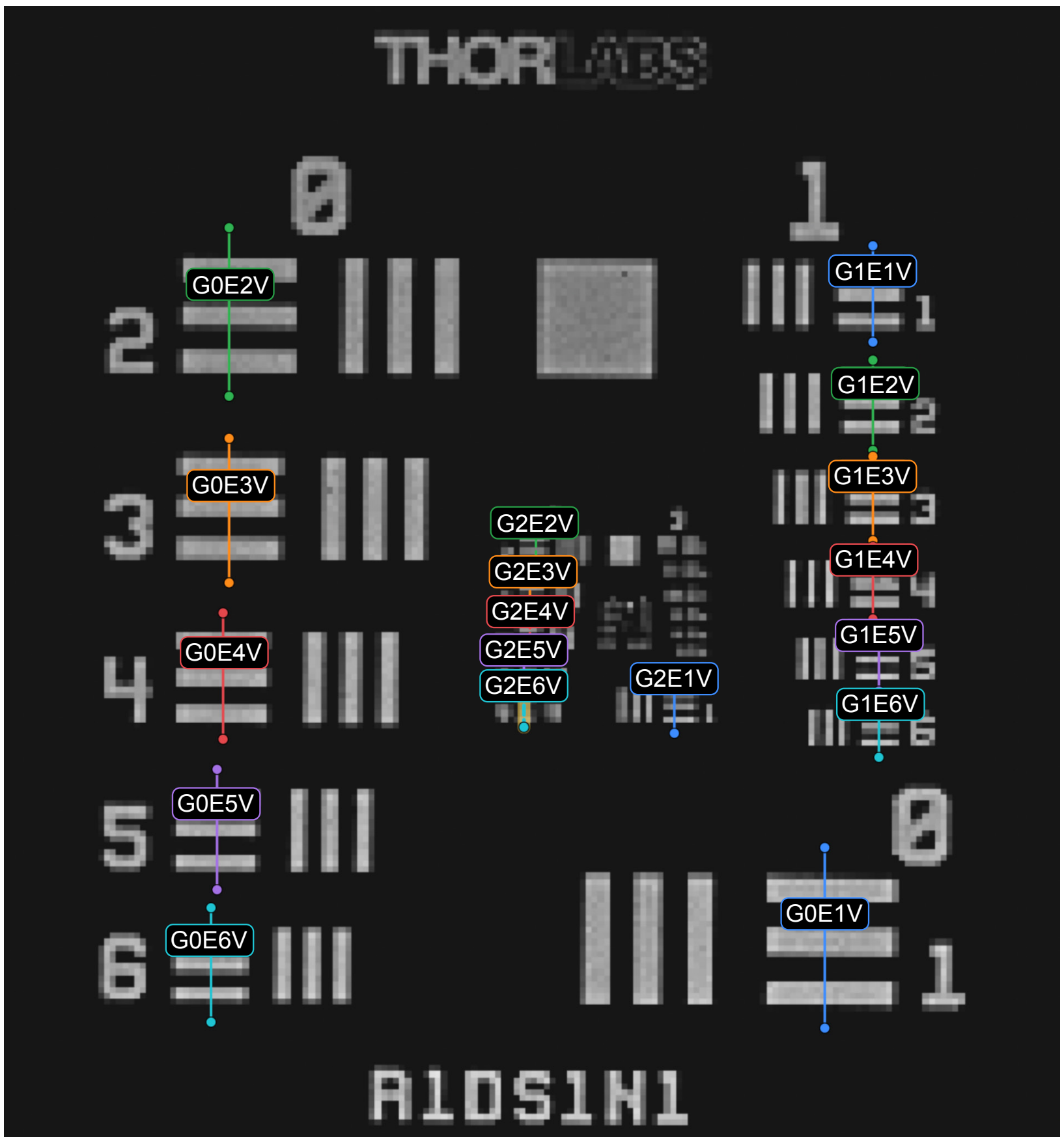

Supplementary Figure S1: Negative USAF 1951 reflection target (Thorlabs R1DS1N1) used for lateral-resolution analysis. Colored line segments indicate representative sampling locations used to extract line profiles for Michelson-contrast measurement.

Supplementary Table S3: Visible-channel lateral-resolution summary under clinically matched white-light imaging conditions.

Channel	Highest passing element	Spatial frequency ($\mathrm{lp\,mm^{-1}}$)	Half-pitch (μm)	Criterion
LG-B	G2E4	5.66	88.3	$\bar{C} \geq 0.20$
LG-G	G2E2	4.49	111.4	$\bar{C} \geq 0.20$
LG-R	G2E4	5.66	88.3	$\bar{C} \geq 0.20$

the bright samples, and let L_1 and L_2 denote the dark samples. The mean bright and dark levels were computed as

$$I_H = \frac{H_1 + H_2 + H_3}{3}, \qquad I_L = \frac{L_1 + L_2}{2}. \tag{S8}$$

The five-point Michelson contrast was then calculated as

$$C_M = \frac{I_H - I_L}{I_H + I_L}. \tag{S9}$$

When multiple vertical profiles were measured for the same group/element, the reported cell value was the mean of all Michelson-contrast measurements for that group/element:

$$\bar{C}(G, E) = \frac{1}{N} \sum_{n=1}^{N} C_{M,n}. \tag{S10}$$

An element was counted as resolved if the mean Michelson contrast satisfied a 20% criterion, that is,

$$\bar{C}(G, E) \geq 0.20. \tag{S11}$$

The limiting resolution for each channel was taken as the highest spatial frequency that still met this threshold. The corresponding minimum resolvable half-pitch in object space was reported as

$$\text{half-pitch} = \frac{1}{2f}\ \text{mm} = \frac{500}{f}\ \mu\text{m}. \tag{S12}$$

S3.3 Visible-channel resolution under clinical visible-light imaging conditions

Visible-channel resolution was measured from the low-gain blue, green, and red channels under white-light illumination. The resulting channel-wise contrast maps are shown in Supplementary Fig. S2, and the red-channel line profiles used as a visible-channel reference are shown in Supplementary Fig. S3. Using the 20% Michelson criterion, the highest passing element was Group 2 Element 4 for the low-gain red and blue channels (5.66 $\mathrm{lp\,mm^{-1}}$), and Group 2 Element 2 for the low-gain green channel (4.49 $\mathrm{lp\,mm^{-1}}$). These values correspond to minimum resolvable half-pitches of 88.3 μm for the red and blue channels and 111.4 μm for the green channel.

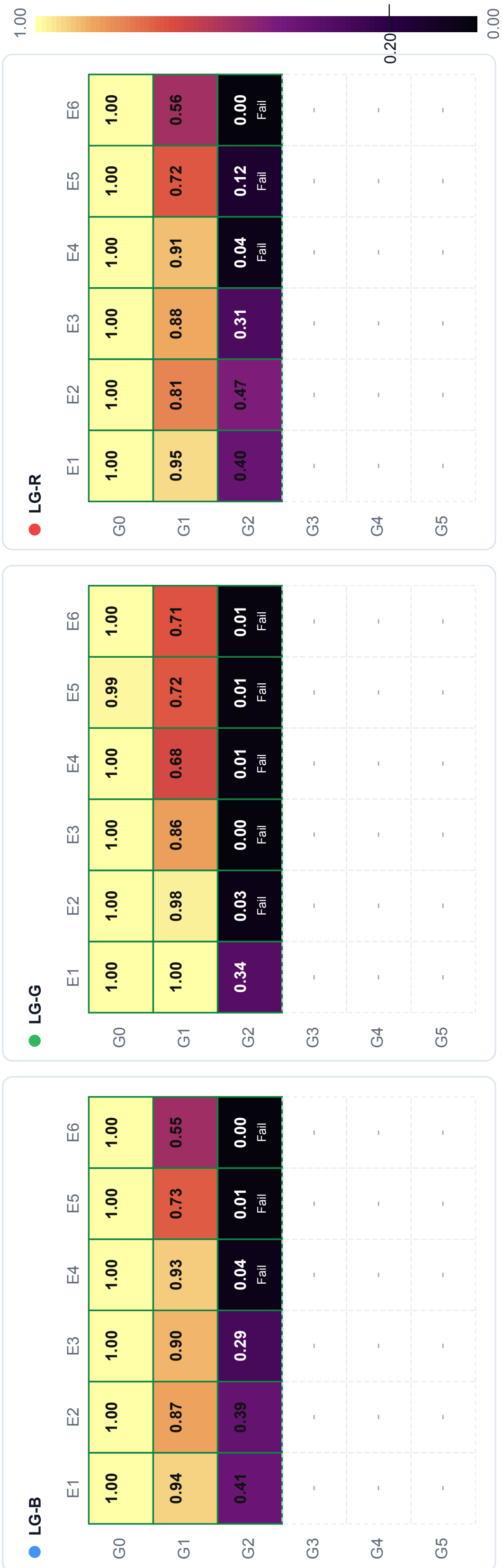

Supplementary Figure S2: Mean five-point Michelson-contrast maps for the low-gain visible channels under white-light illumination. Rows correspond to USAF groups, columns correspond to elements 1–6, and each cell reports the mean contrast over all selected vertical profiles for that group/element. Using a 20% threshold, the limiting visible resolution was 5.66 lp mm^{-1} for the red and blue channels and 4.49 lp mm^{-1} for the green channel.

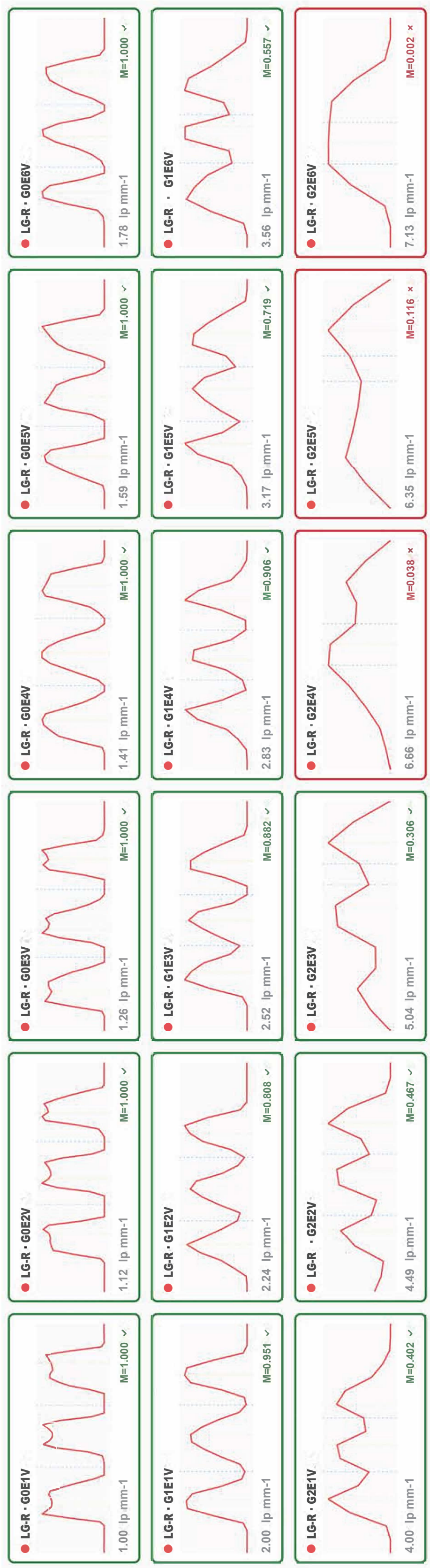

Supplementary Figure S3: Red-channel line profiles used as representative visible-channel traces for the negative USAF target.

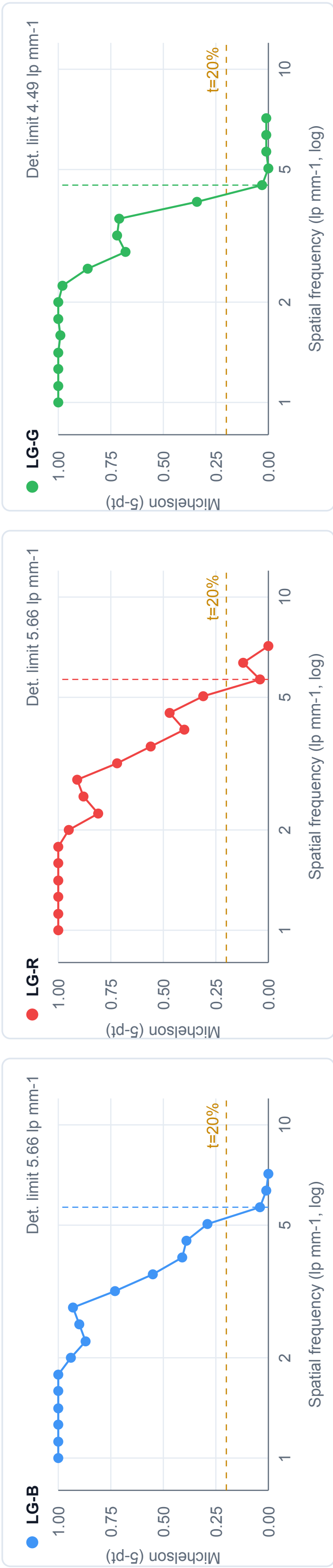

Supplementary Figure S4: Channel-wise Michelson contrast versus spatial frequency for the low-gain visible channels. The dashed horizontal line marks the 20% passing threshold. The limiting frequencies were 5.66 lp mm^{-1} for red and blue and 4.49 lp mm^{-1} for green.

S3.4 NIR-channel resolution under clinical NIR imaging conditions

NIR resolution was measured under 810 nm LED illumination using the high-gain NIR channel, which is the channel used for weak-signal fluorescence imaging in the clinical workflow. Supplementary Fig. S5 through S7 show the mean Michelson-contrast maps for both the high-gain and low-gain NIR channels for completeness. The reported NIR resolution value was taken from the high-gain channel. Using the same 20% threshold, the highest passing element for HG-NIR was Group 2 Element 3, corresponding to 5.04 $\mathrm{lp\,mm^{-1}}$. This corresponds to a minimum resolvable half-pitch of 99.2 μm. Group 2 Element 4 (5.66 $\mathrm{lp\,mm^{-1}}$) fell below the 20% contrast threshold in the high-gain NIR data.

Supplementary Table S4: NIR-channel lateral-resolution summary under clinically matched 810 nm imaging conditions.

Channel	Highest passing element	Spatial frequency ($\mathrm{lp\,mm^{-1}}$)	Half-pitch (μm)	Criterion
HG-NIR	G2E3	5.04	99.2	$\bar{C} \geq 0.20$

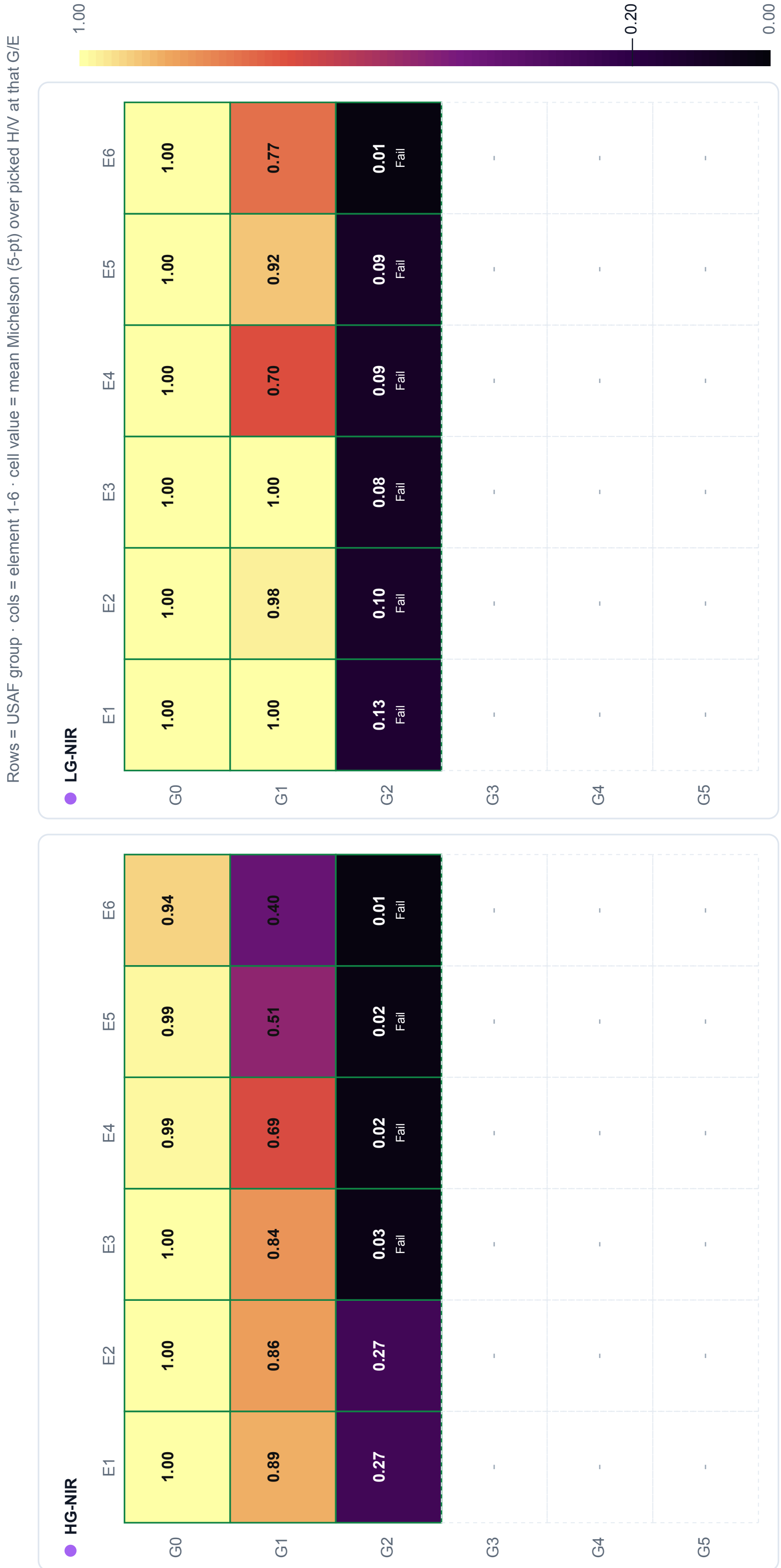

Supplementary Figure S5: Mean five-point Michelson-contrast maps for the NIR channel under 810 nm illumination. High-gain NIR (left) was used for the reported NIR resolution because it is the operative weak-signal NIR readout. Low-gain NIR (right) is shown for completeness.

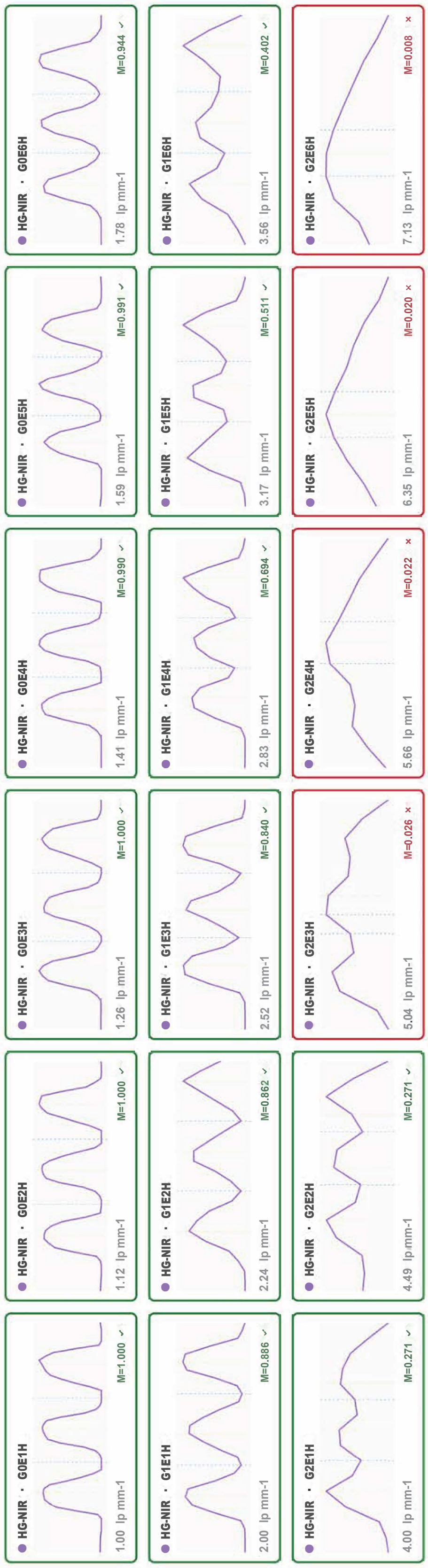

Supplementary Figure S6: High-gain NIR line profiles across all evaluated USAF elements under 810 nm illumination. These traces were used to compute the five-point Michelson contrast reported in Supplementary Fig. S5.

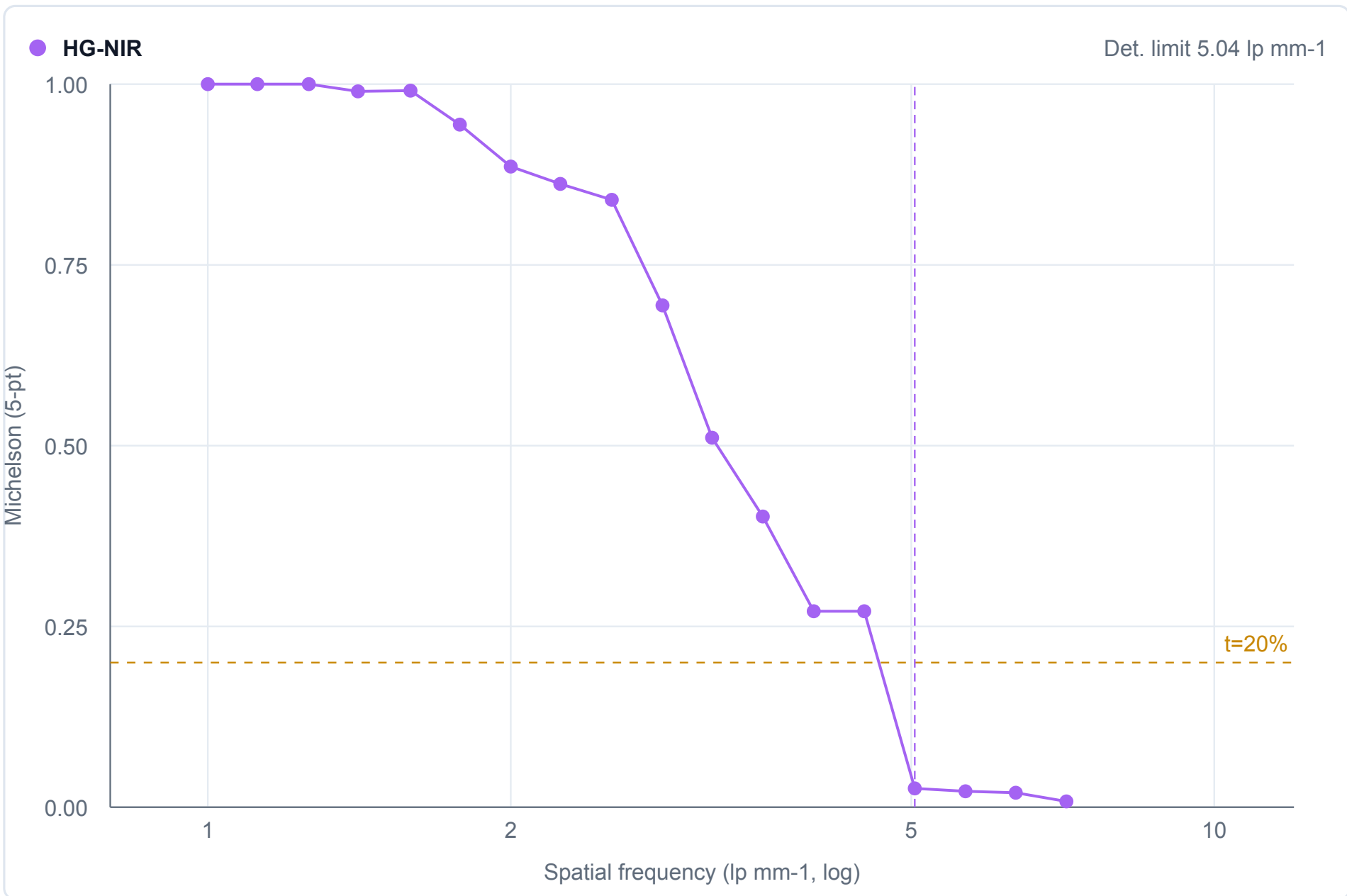

Supplementary Figure S7: High-gain NIR Michelson contrast versus spatial frequency. The dashed horizontal line marks the 20% threshold. The highest passing element was Group 2 Element 3, giving a limiting NIR resolution of 5.04 $\text{lp}\,\text{mm}^{-1}$.

S4 Fixed-pattern noise (FPN) / PRNU characterization

Fixed-pattern noise was evaluated under the same imaging configuration used in the clinical study: the same 40 mm imaging lens (∼90 mm full-frame equivalent on an 18.3 mm diagonal sensor), the same working distance (∼0.5 m), the same f-number (f/8), and the same acquisition pipeline. One spatially uniform frame was acquired at 530 nm for evaluation of the red, green, and blue channels, and one spatially uniform frame was acquired at 800 nm for evaluation of the NIR channel. Both LEDs were delivered through the LED-driven integrating sphere configuration described in main-text Methods Section 5.7.

S4.1 Method of analysis

To characterize photoresponse non-uniformity (PRNU), a representative uniform region of interest (ROI-1) was selected in each channel image. The analysis was performed on the low-gain outputs, which placed the ROI near mid-scale (approximately 40% of the 16-bit range) while avoiding clipping. No flat-field correction was applied before calculating PRNU, so the reported values reflect the raw residual spatial non-uniformity of the imaging chain under clinically matched optics.

For a given channel image $I(x, y)$ defined on an ROI Ω containing N pixels, the mean signal μ and spatial standard deviation σ were computed as

$$\mu = \frac{1}{N} \sum_{(x,y)\in\Omega} I(x, y), \qquad \sigma = \sqrt{\frac{1}{N-1} \sum_{(x,y)\in\Omega} \big(I(x, y) - \mu\big)^2}. \tag{S13}$$

The PRNU was then reported as

$$\text{PRNU}\,(\%) = 100 \times \frac{\sigma}{\mu}. \tag{S14}$$

For visualization, row-wise and column-wise mean profiles were also computed to show the spatial structure of the residual non-uniformity:

$$r(i) = \frac{1}{W} \sum_{j} I(i, j), \qquad c(j) = \frac{1}{H} \sum_{i} I(i, j), \tag{S15}$$

where W and H are the ROI width and height, respectively. Histogram plots were additionally generated after normalizing the raw 16-bit pixel values to full scale,

$$\frac{\text{pixel value}}{65535} \times 100\%. \tag{S16}$$

These histograms are included only to show distribution shape and spread; the reported PRNU values were computed directly from the raw digital numbers.

S4.2 Results

The measured PRNU values for the four low-gain channels were 1.395% (green), 1.321% (red), 1.513% (blue), and 1.185% (NIR), corresponding to an average PRNU of 1.35% and a maximum PRNU of 1.51%. Across channels, the ROI means were centered near 40% of full scale, indicating that the comparison was performed at similar signal levels. The row/column traces show that the residual non-uniformity was dominated by smooth low-frequency structure rather than by strong row-wise striping or localized defects.

Supplementary Table S5: Summary of PRNU measurements in a representative ROI (ROI-1).

**Channel**	**Mean (DN)**	**Mean (%FS)**	σ **(DN)**	**PRNU (%)**	$\sigma_{\mathbf{row}}$ **(DN)**	$\sigma_{\mathbf{col}}$ **(DN)**
LG-G	26784.9	40.9	373.54	1.395	29.512	311.486
LG-R	26237.0	40.0	346.59	1.321	23.740	272.148
LG-B	25864.8	39.5	391.32	1.513	34.355	329.401
LG-NIR	27310.0	41.7	323.71	1.185	24.837	252.817
Average / max	—	—	—	1.35 / 1.51	—	—

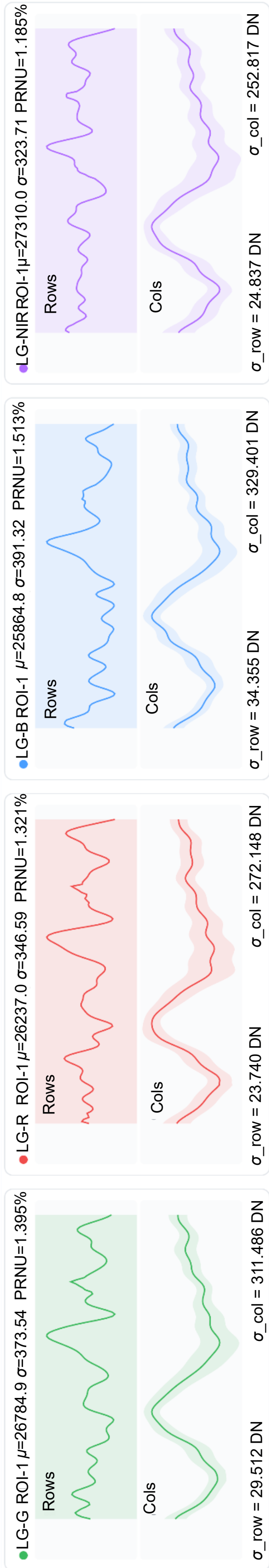

Supplementary Figure S8: Representative row-wise and column-wise mean profiles for ROI-1 in the four low-gain channels. Each panel reports the ROI mean μ, spatial standard deviation σ, and PRNU $= 100\sigma/\mu$. The small σ_{row} values relative to σ_{col} indicate that the residual spatial non-uniformity is dominated by smooth column-direction structure rather than strong row striping.

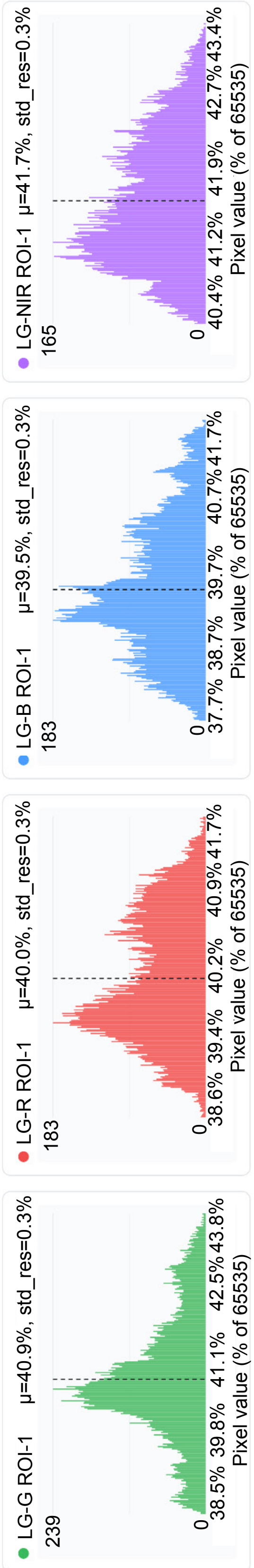

Supplementary Figure S9: Histograms of ROI-1 pixel values after normalization to the 16-bit full-scale range. The distributions are centered near 40% of full scale for all channels, with residual spread on the order of 0.3% of full scale, consistent with the low PRNU values reported above.

S5 Depth of focus measurement

All depth-of-focus measurements were acquired under the same optical configuration used in the clinical study: the same 40 mm imaging lens (approximately 90 mm full-frame equivalent on the 18.3 mm diagonal sensor), the same working distance (∼0.5 m), the same aperture setting (f/8), and the same sensor operating parameters used during clinical imaging. Visible-channel measurements used the same broadband visible LED source used in the resolution study (Thorlabs MNWHL4, 4900 K), and NIR measurements used the same NIR LED source (Thorlabs M810L5, 810 nm).

S5.1 Depth-of-field target, geometry, and calibration

Depth of focus (DoF) was measured with the Edmund Optics Depth of Field Target 5–15. The target was placed on a flat support and tilted by 45° with respect to the image plane, while the camera viewed the target from above. Because the target contains a known rectilinear geometry, the imaged corners and feature boundaries were used to calibrate a planar perspective transform (homography), allowing pixel coordinates to be mapped to metric coordinates on the target surface. A line of analysis was then defined along the target's depth-varying direction so that each image position corresponded to a known physical position along the tilted target:

$$[x_t,\ y_t,\ 1]^\mathsf{T} \propto H\,[u,\ v,\ 1]^\mathsf{T}. \tag{S17}$$

After perspective correction, position along the target, denoted s (mm), was converted to the axial defocus coordinate z using the known target tilt angle $\theta = 45°$:

$$z(s) = (s - s_0)\sin\theta, \tag{S18}$$

where s_0 is an arbitrary reference position on the target. In practice, the focus curve was evaluated in the calibrated physical domain, so the fitted width directly corresponds to the camera depth of field (DoF) under the stated imaging condition.

S5.2 Composite focus score

At each position along the calibrated target, four standard sharpness measures were evaluated: the Laplacian peak $L(z)$, the Brenner peak $B(z)$, the Tenengrad peak $T(z)$, and the high-frequency peak $F(z)$ of the Fourier magnitude spectrum. Each metric was normalized to its own maximum across the sweep and then combined into a single composite focus score:

$$\tilde{L}(z_i) = \frac{L(z_i)}{\max_z L(z)}, \tag{S19}$$

$$\tilde{B}(z_i) = \frac{B(z_i)}{\max_z B(z)}, \tag{S20}$$

$$\tilde{T}(z_i) = \frac{T(z_i)}{\max_z T(z)}, \tag{S21}$$

$$\tilde{F}(z_i) = \frac{F(z_i)}{\max_z F(z)}, \tag{S22}$$

$$S(z_i) = \frac{\tilde{L}(z_i) + \tilde{B}(z_i) + \tilde{T}(z_i) + \tilde{F}(z_i)}{4}. \tag{S23}$$

Averaging multiple focus measures reduces metric-specific bias and yields a smoother, more robust estimate of the in-focus region than any single sharpness score alone.

S5.3 Gaussian fit and DoF extraction

The composite focus curve $S(z)$ was fitted with a Gaussian model with constant baseline:

$$S(z) = c + A\,\exp\!\left(-\frac{(z-\mu)^2}{2\sigma^2}\right), \tag{S24}$$

where A is the peak amplitude, μ is the best-focus position, σ is the fitted standard deviation, and c is a constant baseline offset. The coefficient of determination R^2 was used to assess the goodness of fit. Depth of field (DoF) was defined from the full width at half maximum (FWHM) of the fitted Gaussian:

$$\mathrm{FWHM} = 2\sqrt{2\ln 2}\,\sigma. \tag{S25}$$

When the fit is performed in the calibrated physical depth domain, the FWHM gives the depth of focus directly. The fitted Gaussian and the resulting DoF were computed independently for the red, green, blue, and NIR channels.

S5.4 Measured depth of focus under clinically matched imaging conditions

Under the clinically matched imaging geometry described above, the visible channels showed DoF values on the order of 27–30 mm, while the NIR channel showed a somewhat smaller DoF. The representative HG-channel fit outputs yielded DoF values of 28.776 mm (R), 27.084 mm (G), 27.084 mm (B), and 23.698 mm (NIR). For the final summary, these values were rounded and reported as 28.8 mm (R), 27.1 mm (G), 27.1 mm (B), and 23.7 mm (NIR). These values are consistent with the expected optical behavior at f/8 and indicate that the system retains a clinically useful tolerance to target height variation across both visible and NIR imaging modes.

Supplementary Table S6: Summary of measured depth of focus under clinically matched imaging conditions.

**Channel**	**Illumination**	**Reported DoF (mm)**
R	Thorlabs MNWHL4 (4900 K)	28.8
G	Thorlabs MNWHL4 (4900 K)	27.1
B	Thorlabs MNWHL4 (4900 K)	27.1
NIR	Thorlabs M810L5 (810 nm)	23.7

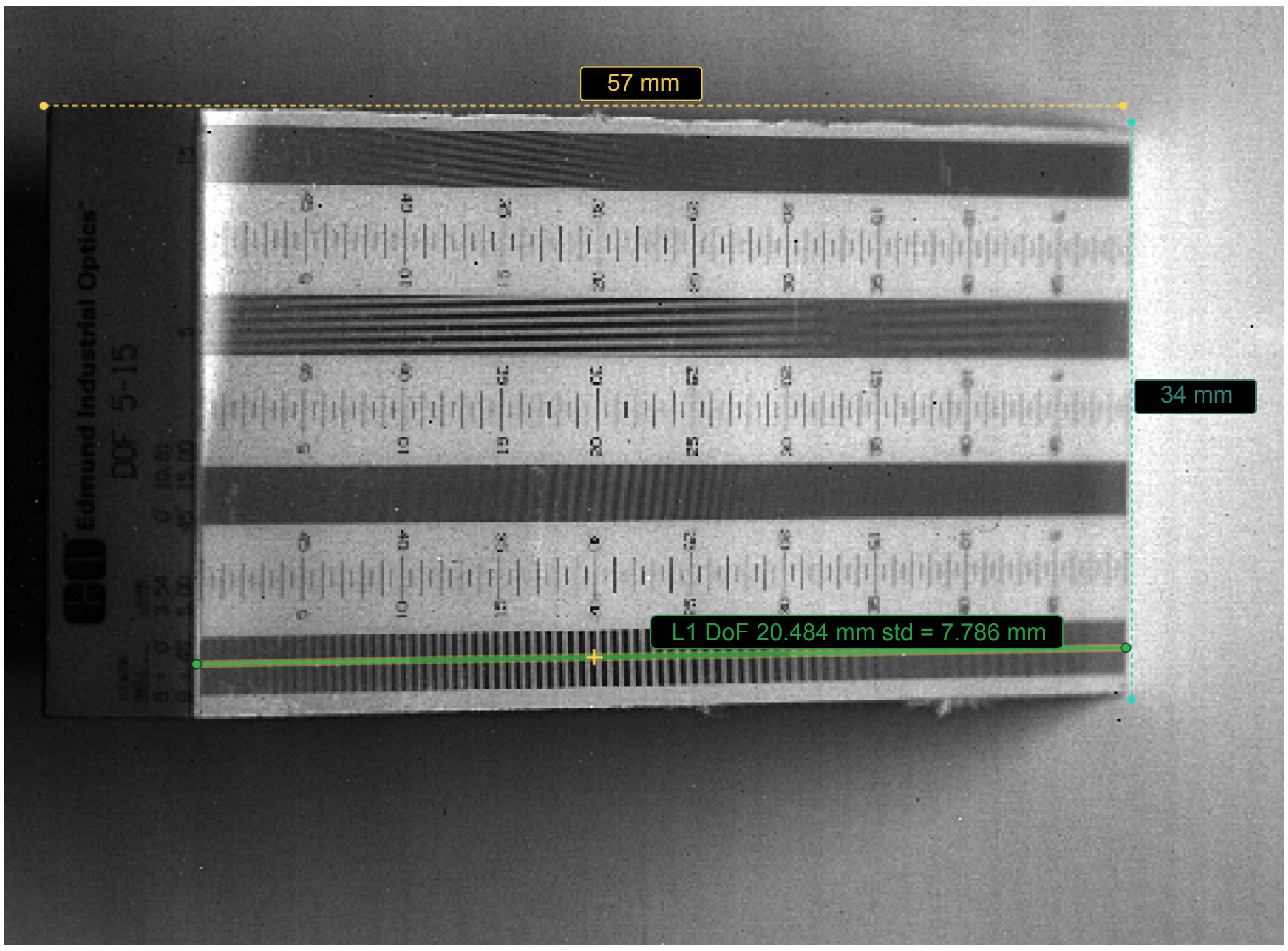

Supplementary Figure S10: Representative acquisition of the Edmund Optics Depth of Field Target 5–15 under the clinically matched optical setup. The target was placed at 45° relative to the image plane and imaged from above. The known target geometry was used for perspective calibration, and the analysis line along the tilted axis was converted from image coordinates to physical position for subsequent focus-curve fitting.

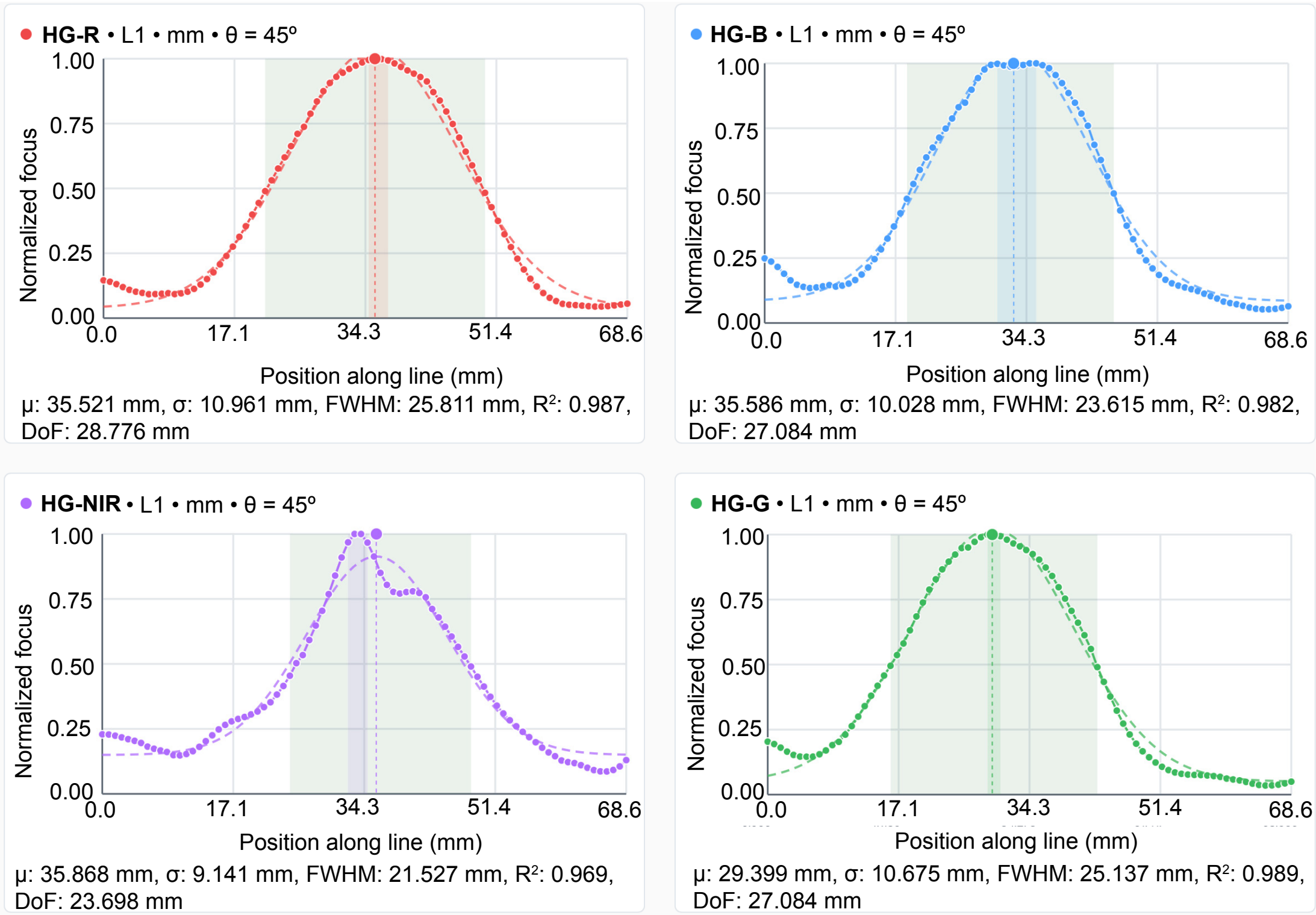

Supplementary Figure S11: Per-channel Gaussian-fit analysis of the composite focus score for the red, green, blue, and NIR channels. Filled markers denote the normalized composite focus score derived from the mean of the normalized Laplacian, Brenner, Tenengrad, and FFT high-frequency peaks. Solid curves denote the fitted Gaussian-with-baseline model. The fitted FWHM in the calibrated depth domain was used to report depth of focus. Representative fitted DoF values from these HG-channel plots were 28.776 mm (R), 27.084 mm (G), 27.084 mm (B), and 23.698 mm (NIR).

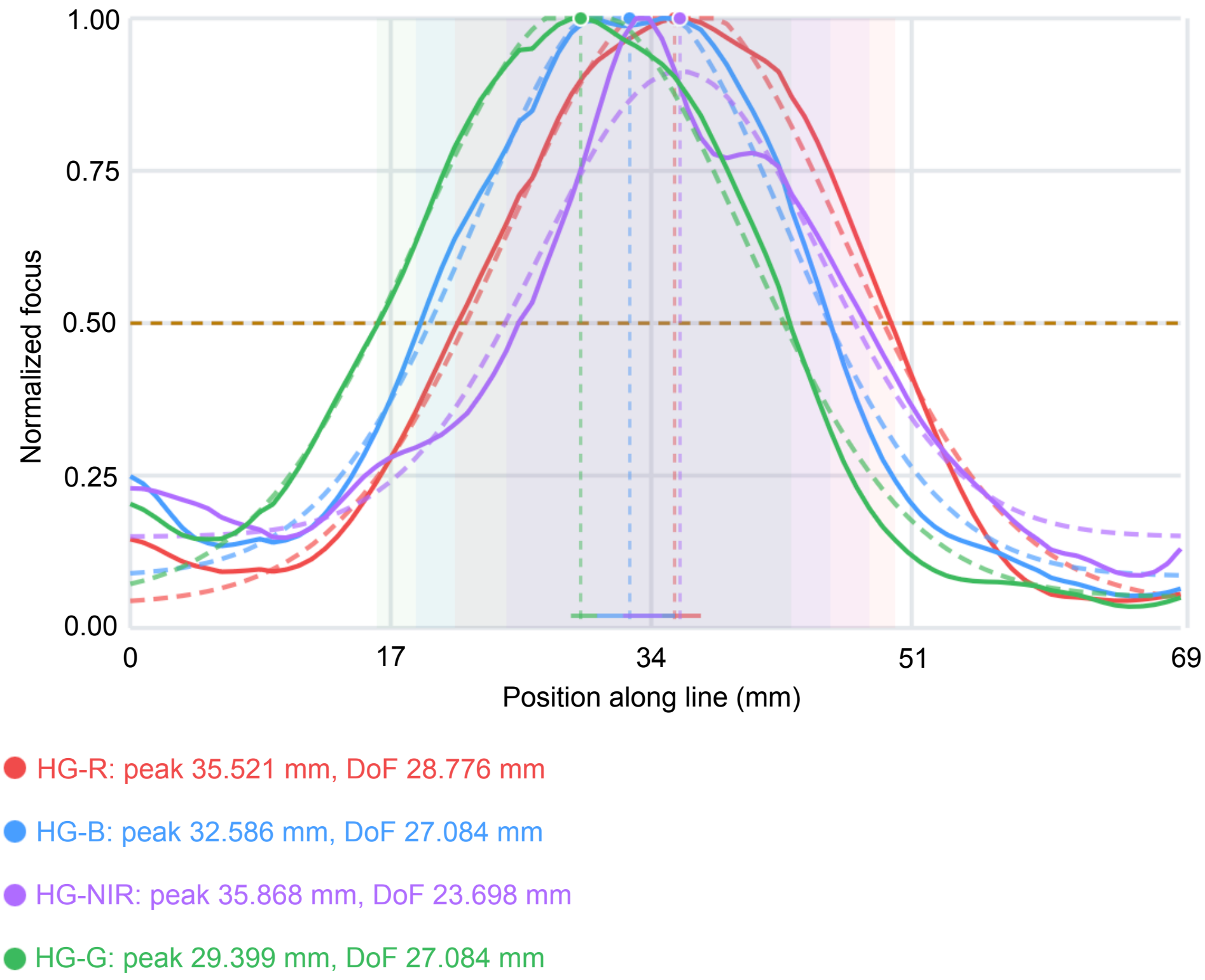

Supplementary Figure S12: All-channel fit overlay. The plot shows the normalized composite focus score, the fitted Gaussian-with-baseline model, the best-focus position μ, the fitted width σ, the derived FWHM, and the resulting DoF.

S6 ICG-equivalent depth-sensitivity assessment

As discussed in the main text, effective fluorescence tissue-imaging depth is governed by the combined effects of fluorophore brightness and concentration, tissue absorption and scattering, excitation irradiance, optical collection geometry, and the detection threshold used to define visibility, rather than by the camera alone. To complement that discussion with a reproducible sensor-side benchmark, we additionally evaluated the prototype against a standardized ICG-equivalent depth-sensitivity phantom under the same imaging conditions used for all other optical-performance measurements and the clinical study.

S6.1 Phantom and imaging conditions

The phantom was a commercial ICG-Equivalent Depth Sensitivity Target (QUEL Imaging, model V2_R3-03). The target consists of an array of fluorescent wells embedded at calibrated depths within a tissue-mimicking matrix whose NIR optical properties are matched to ICG. The version used here contains eight wells at depths from approximately 0.5 mm to 6 mm. Throughout this section, well #1 denotes the deepest well (∼6 mm) and well #8 denotes the shallowest well (∼0.5 mm).

Imaging conditions were identical to those used for all other optical-performance and clinical measurements: 785 nm laser excitation delivered at an irradiance of 20 $\mathrm{mW\,cm^{-2}}$, the same 40 mm imaging lens (approximately 90 mm full-frame equivalent on the 18.3 mm diagonal sensor), the same working distance (∼0.5 m), the same aperture (f/8), and the same dual-gain readout pipeline. Example color and NIR images acquired with our sensor are shown in Supplementary Fig. S13.

S6.2 Analysis

For each of the eight wells, a region of interest (ROI) was drawn over the well and a matched background ROI was drawn over an adjacent non-well region of the tissue-mimicking matrix at the same nominal depth. Baseline ratio was defined as the mean dark-subtracted NIR-channel signal in the well ROI divided by the mean dark-subtracted NIR-channel signal in the matched background ROI:

$$\text{Baseline ratio} = \frac{\mu_{\text{well}}}{\mu_{\text{background}}}. \tag{S26}$$

Standard deviation was propagated from the per-pixel statistics of the two ROIs. A baseline ratio greater than unity indicates that the well is distinguishable from its adjacent matrix; the dashed amber line in Supplementary Fig. S14 marks this no-contrast threshold.

S6.3 Results

All eight wells exceeded the no-contrast threshold under the stated imaging conditions, corresponding to 100% of evaluated depths showing measurable contrast (Supplementary Fig. S14). Across the eight wells, the mean baseline ratio was 2.86 (±0.76 SEM; 95% CI 1.37–4.35), the median was 1.97, the interquartile range was 1.39–2.70, and the minimum and maximum were 1.36 and 7.80, respectively. The standard deviation across wells was 2.15, reflecting the expected reduction of contrast with increasing well depth rather than measurement variability at a fixed depth.

Baseline ratio decreased monotonically with increasing well depth, from approximately 7.80 at the shallowest well (#8) to 1.36 at the deepest 6 mm well (#1) (Supplementary Fig. S15). The deepest well still produced contrast above the no-contrast threshold under the prototype's clinical imaging configuration, indicating that all phantom depths available in the V2_R3-03 target were resolvable by the sensor under the stated optical conditions.

We emphasize that this phantom benchmark characterizes sensor-side performance against a defined tissue-optics target and is not equivalent to a single, generalized in-tissue depth value, because actual in-tissue depth depends on probe pharmacokinetics, local fluorophore concentration, regional tissue optical properties, and the detection threshold used to define visibility.

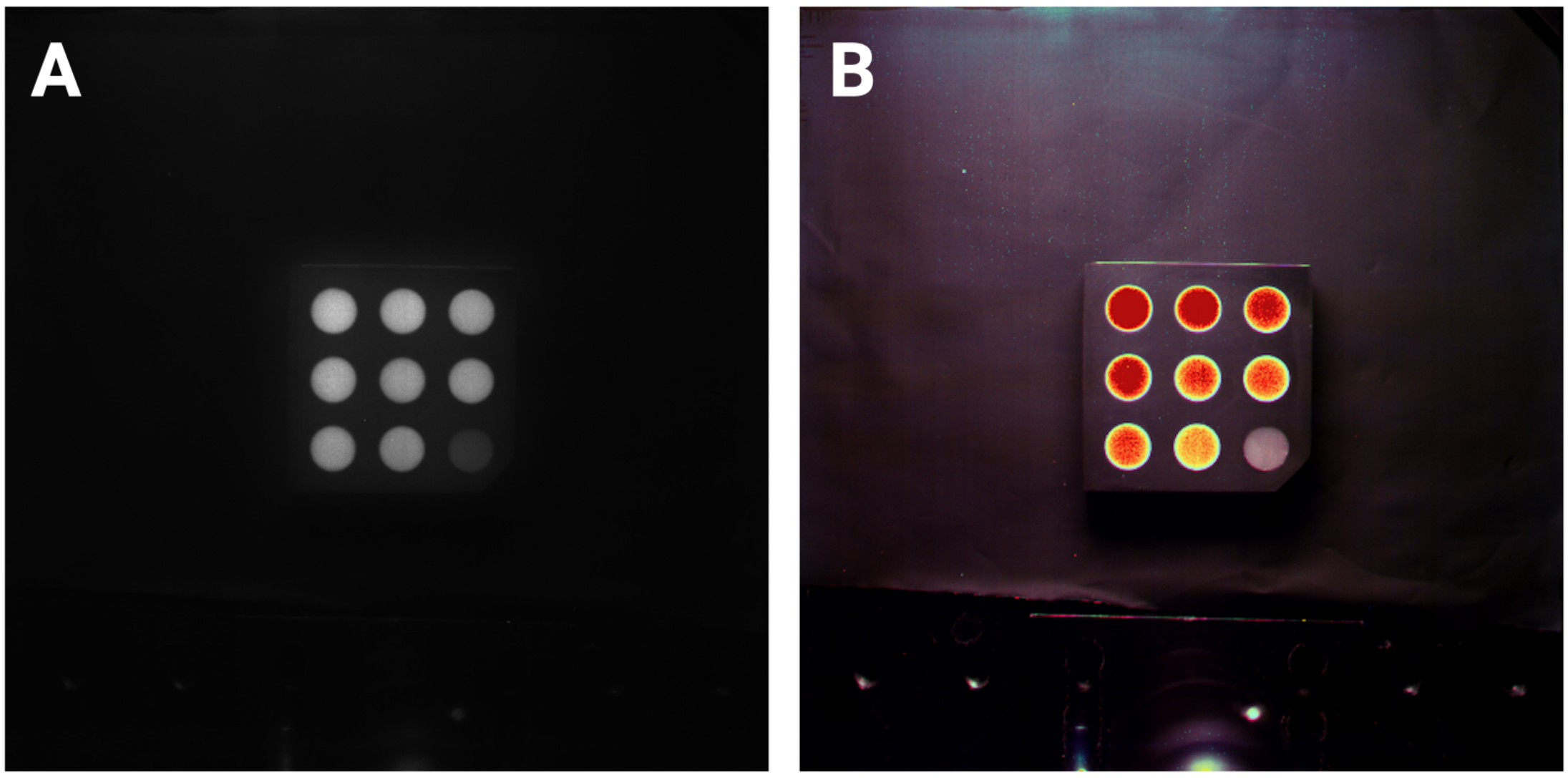

Supplementary Figure S13: Direct view of the QUEL Imaging V2_R3-03 ICG-equivalent depth-sensitivity phantom acquired by the prototype RGB–NIR camera under 785 nm excitation at 20 $\mathrm{mW\,cm^{-2}}$, with the same lens, working distance, aperture, and dual-gain readout pipeline used for all other optical-performance and clinical measurements. (A) Raw high-gain NIR-channel image of the 3 $\times$ 3 well array. Eight wells contain ICG-equivalent fluorophore at calibrated depths up to 6 mm in a tissue-mimicking matrix; the bottom-right position is a non-fluorescent reference well. The deepest fluorescent well ($\sim$6 mm) is just distinguishable above the matrix background, consistent with the baseline ratio of 1.36 measured at that depth (Supplementary Fig. S14, well #1). (B) Fused HDR overlay of the same NIR-channel data on the simultaneously acquired visible-channel image of the same field of view, demonstrating intrinsic spatial coregistration between fluorescence signal and anatomical context. Both panels were captured from a single dual-gain exposure (Supplementary Section S1); the non-fluorescent reference well at the bottom-right appears as visible-channel context in (B) without a fluorescence overlay.

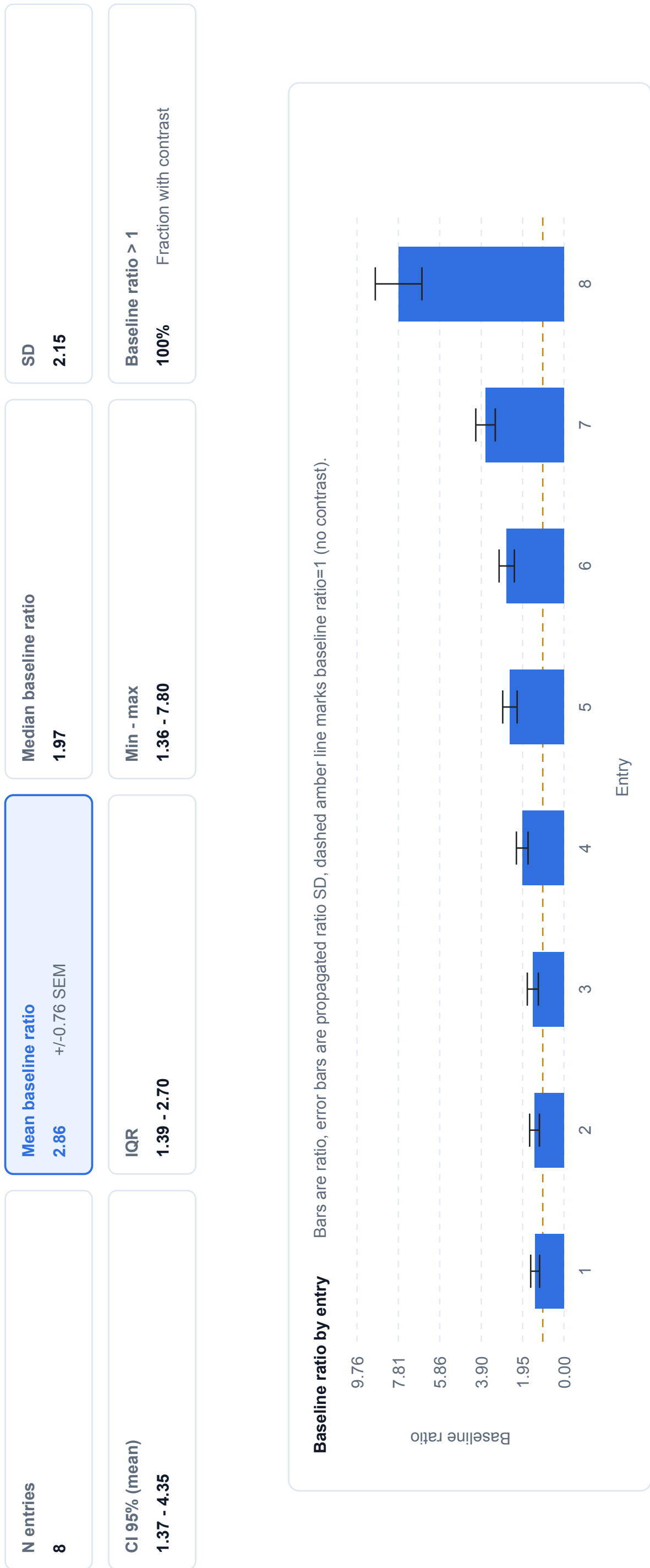

Supplementary Figure S14: Baseline ratio for each well in the standardized ICG-equivalent depth-sensitivity phantom (QUEL Imaging, V2_R3-03), measured with the prototype RGB–NIR camera under 785 nm excitation at 20 $\mathrm{mW\,cm^{-2}}$ and the same imaging configuration used for all other optical-performance and clinical measurements. Bars are the mean baseline ratio per well; error bars are the propagated standard deviation. The dashed amber line marks Baseline Ratio = 1 (no contrast against the adjacent matrix). All eight wells exceed the no-contrast threshold (100% of wells with contrast). Across the eight wells, the mean baseline ratio is 2.86 (±0.76 SEM; 95% CI 1.37–4.35), the median is 1.97, the interquartile range is 1.39–2.70, and the minimum and maximum are 1.36 and 7.80, respectively. Wells are indexed so that #1 corresponds to the deepest well (∼6 mm) and #8 to the shallowest well (∼0.5 mm); on this axis the bars are ordered by increasing baseline ratio, which corresponds to decreasing well depth.

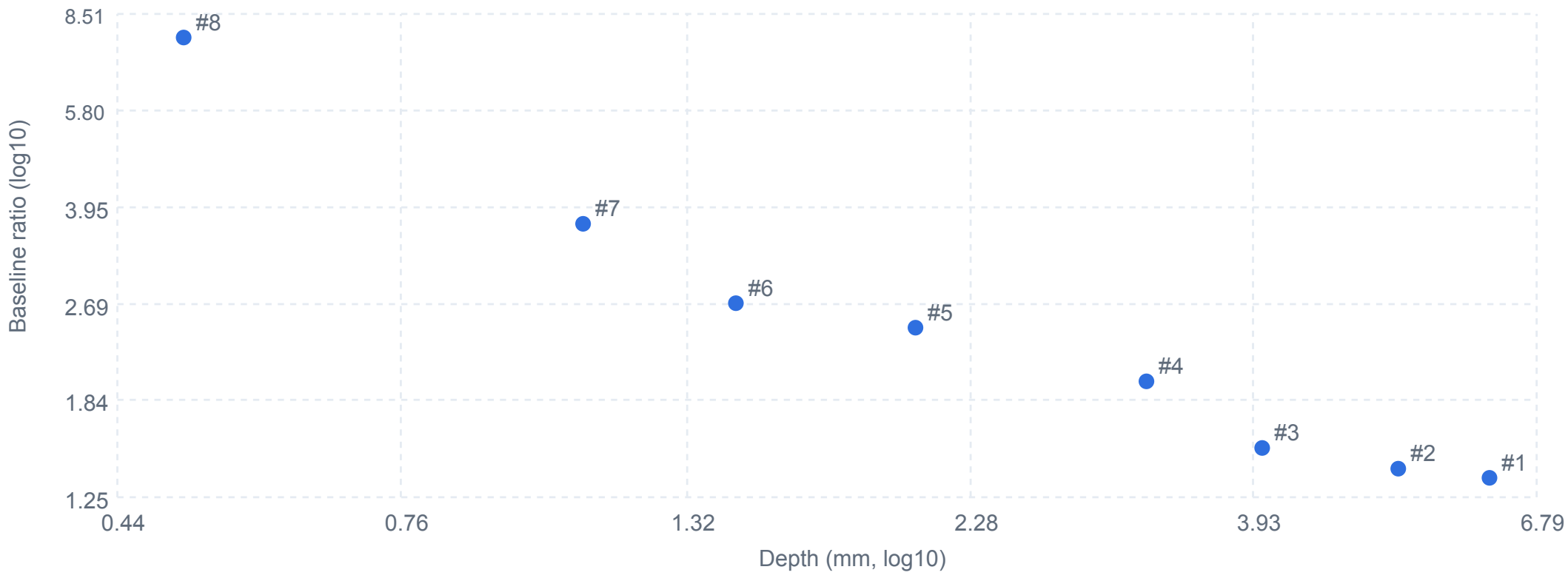

Supplementary Figure S15: Baseline ratio ($\log_{10}$) versus well depth ($\log_{10}$ mm) for the eight calibrated wells in the QUEL ICG-equivalent depth-sensitivity phantom. Baseline ratio decreases monotonically with increasing depth, from ~7.80 at the shallowest well (#8, ~0.5 mm) to 1.36 at the deepest well (#1, ~6 mm). All eight points lie above unity, indicating preserved contrast across the full measurable depth range of the phantom under the prototype's clinical imaging configuration (785 nm, 20 $\mathrm{mW\,cm^{-2}}$).

Supplementary References

[1] D'Souza, A. V., Lin, H., Henderson, E. R., Samkoe, K. S. & Pogue, B. W. Review of fluorescence guided surgery systems: Identification of key performance capabilities beyond indocyanine green imaging. *Journal of Biomedical Optics* **21**, 080901 (2016).

[2] Stryker. SPY-PHI Fluorescence Imaging Operator's Manual. Stryker product literature (2024). NIR laser-diode excitation at 805 nm; CMOS HD sensor.

[3] U.S. Food and Drug Administration. SPY AGENT GREEN prescribing information / label. Includes breast lymphatic mapping indication (2023). 510(k) summary describes the open-surgery ICG predicate platform.

[4] onsemi. AR0830: 1/2.9-inch 8 MP CMOS Digital Image Sensor (2026). URL https://www.onsemi.com/download/public-data-sheet/pdf/ar0830-pd-d.pdf. Public data sheet, Rev. 16.

[5] e-con Systems. See3CAM_CU83: 8MP 4K RGB-IR USB 3.1 Gen 1 Camera. URL https://www.e-consystems.com/usb-cameras/8mp-4k-rgb-ir-usb3-camera.asp.

[6] OMNIVISION. OV2312 1/2.9-inch RGB-IR 2-Megapixel CMOS Image Sensor Product Brief (2023). URL https://www.ovt.com/wp-content/uploads/2023/07/OV2312-PB-v1.2-WEB.pdf. Version 1.2.

[7] e-con Systems. See3CAM_27CUG: 2MP OV2312 Global Shutter RGB-IR Camera. URL https://www.e-consystems.com/usb-cameras/2mp-ov2312-global-shutter-rgb-ir-camera.asp.

[8] OMNIVISION. OX05B1S 5-Megapixel Product Brief (2025). URL https://www.ovt.com/wp-content/uploads/2025/10/OX05B1S-PB-v1.2-WEB.pdf. Version 1.2.

[9] e-con Systems. STURDeCAM57: 5MP OX05B1S RGB-IR Global Shutter GMSL2 Camera. URL https://www.e-consystems.com/gmsl-cameras/5mp-ox05b1s-rgb-ir-global-shutter-gmsl2-camera.asp.

[10] ams OSRAM. Mira220 1/2.7-inch 2.2 MP NIR Enhanced Global Shutter Image Sensor (2025). URL https://look.ams-osram.com/m/7591efdbe4af32dc/original/Mira220-1-2-7-2-2-MP-NIR-enhanced-global-shutter-image-sensor.pdf. Public datasheet DS000642, v9-00.

[11] Arducam. 2.2MP Mira220 RGB-IR Global Shutter USB3.0 Camera Evaluation Kit. URL https://www.arducam.com/arducam-2-2mp-mira220-rgb-ir-global-shutter-usb3-0-camera-evaluation-kit.html.

[12] ams OSRAM. CMV4000 Global Shutter CMOS Image Sensor for Machine Vision (2025). URL https://look.ams-osram.com/m/5ddf0ec561bcbdcc/original/CMV4000-Global-shutter-CMOS-image-sensor-for-machine-vision.pdf. Public datasheet DS000728, v8-01.

[13] Spectral Devices. MSC2-RGBN-1-A RGB-NIR Camera. URL https://spectraldevices.com/products/rgb-nir-camera.

[14] Chen, Z., Zhu, N., Pacheco, S., Wang, X. & Liang, R. Single camera imaging system for color and near-infrared fluorescence image guided surgery. *Biomedical Optics Express* **5**, 2791–2797 (2014).

[15] Garcia, M. *et al.* Bio-inspired imager improves sensitivity in near-infrared fluorescence image-guided surgery. *Optica* **5**, 413–422 (2018).

[16] Blair, S. *et al.* Hexachromatic bioinspired camera for image-guided cancer surgery. *Science Translational Medicine* **13**, eaaw7067 (2021).

[17] Gpixel. GSENSE2020BSI 6.5 μm, 4 mp scientific bsi CMOS image sensor. Product datasheet, version 1.1.3 (2021). HDR image construction guidance reproduced from the manufacturer datasheet.
